# Monocarboxylate permease gene *SsMP1* regulates *Sporisorium scitamineum* mating, pathogenicity and absorption and transport of citric acid

**DOI:** 10.3389/fmicb.2026.1826158

**Published:** 2026-07-08

**Authors:** Sisi Zhou, Yi Zhang, Jiali Zhou, Xiaohui Huang, Wankuan Shen

**Affiliations:** 1 College of Agriculture, South China Agricultural University, Guangzhou, China; 2 Sugarcane Research Laboratory, South China Agricultural University, Guangzhou, China

**Keywords:** gene complementarity, gene knockout, monocarboxylate permease, pathogenicity, sugarcane smut

## Abstract

Sugarcane is an important sugar crop, and smut disease caused by *Sporisorium scitamineum* poses a severe threat with limited control measures. This study is based on transcriptome sequencing data from two distinct pathogenic strains of *Sporisorium scitamineum* generated in our laboratory. Among the significantly differentially expressed genes, we identified a gene predicted to encode a monocarboxylate permease, which we designated *SsMP1*. To investigate its biological function, *SsMP1* knockout and complemented mutants were generated using PEG-mediated protoplast transformation. Our results showed that *SsMP1* had little to no effect on sporidial morphology, colony morphology, growth rate, or tolerance to abiotic stresses. In the knockout mutant, *SsMP1* expression was undetectable, whereas it was readily detected in both the wild-type and complemented strains, Moreover, the expression level in the complemented mutant was restored to that of the wild-type strain. Notably, sexual mating ability was almost completely abolished in the knockout mutant but was fully restored in the complemented mutant. Interestingly, supplementation with exogenous signaling molecules, including cAMP or tryptophol, largely rescued the mating defect of the knockout mutant. Consistently, the expression levels of *Uac1*, a gene involved in cAMP biosynthesis, and *Aro8*, a gene associated with tryptophol biosynthesis, were significantly lower in the knockout mutant than in the wild-type and complemented strains. In addition, the knockout mutant exhibited more than a 70% reduction in pathogenicity compared with the wild-type and complemented strains. Furthermore, citric acid transport and uptake were markedly impaired in the knockout mutant. Taken together, these findings suggest that *SsMP1* may indirectly regulate the expression of key genes involved in the cAMP and tryptophol biosynthetic pathways, thereby modulating signaling molecule production and subsequently affecting mating and pathogenicity. In addition, *SsMP1* appears to positively regulate citric acid uptake and transport in haploid sporidia. This study provides new molecular insights into the pathogenic mechanisms of *S. scitamineum*.

## 1 Introduction

Sugarcane (*Saccharum* hybrids spp.) is an important sugar crop in China. Currently, China ranks as the world’s third-largest sugarcane-producing country, with cultivation primarily distributed in Guangxi, Yunnan, Guangdong, and Hainan provinces (Sica et al., 2023; Li and Yang, 2015; France et al., 2024). The pathogen *Sporisorium scitamineum*, which causes sugarcane smut, was first reported in 1,877 in Natal, South Africa (McMartin, 1945). After infection, sugarcane grows more slowly, with early symptoms including slender leaves and a light green appearance. Infected plants exhibit thinner stalks, increased tillering, and a marked decrease in sucrose content. The most characteristic symptom is the formation of a downward-curled black whip-like structure at the tip of the infected sugarcane (Huang et al., 2018; Marques et al., 2017; Que et al., 2014; Tan and Li, 2015). The disease is more severe in perennial ratoon sugarcane than in newly planted sugarcane (Riley et al., 1999). Over the past 20 years, 90% of China’s sugarcane has been cultivated on dry hillsides, a shift that favors the spread of teliospores from the smut fungus. Consequently, sugarcane smut has become increasingly serious. Currently, most major sugarcane varieties are susceptible to smut disease. The field incidence of the disease ranges from 10 to 20%, and in severely affected fields, the incidence can exceed 50%, sometimes leading to complete crop failure (Tan and Li, 2015). Sugarcane is a highly heterozygous plant with a complex genome and poorly characterized genetic architecture. In addition, its breeding cycle is long, and the existence of many physiological races of *S. scitamineum* makes it difficult to breed smut-resistant sugarcane varieties. Furthermore, teliospores of *S. scitamineum* invade sugarcane buds and spread to the growing points via intercellular hyphae, causing alterations at the growing points and eventually producing black whips. Because sugarcane stalks are high in fiber and wax, conventional chemical agents have difficulty penetrating the stalk tissue and eliminating the pathogen, making disease control particularly challenging (Tan and Li, 2015). Therefore, further research on the pathogenic mechanisms of *S. scitamineum*, especially its molecular pathogenic mechanisms, is necessary to identify new targets for disease control and to develop novel prevention and management strategies.

*S. Scitamineum* belongs to the smut genus of the family *Ustilaginaceae* in the *Subphylum basidiomycota*. This pathogen can parasitize the host and infect crops such as sugarcane and white fescue (McMartin, 1945). *S. scitamineum* is a classic dimorphic fungus possessing two mating types, “+” and “−.” Haploid spores propagate by budding, creating yeast-like colonies, and yield cigar-shaped spores that are non-pathogenic (Dutheil et al., 2016; Barnabas et al., 2016). Only haploid spores of opposite mating types though sexual mating to form dikaryotic hyphae, which are pathogenic and can infect plant tissues (Albert and Schenck, 1996; Sundar et al., 2012). Therefore, the sexual mating of two haploid spores with opposite mating types and the formation of dikaryotic hyphae are critical factors in the pathogenesis of *S. scitamineum* (Li et al., 2021a). In recent years, with the establishment of genetic transformation technology of *S. scitamineum*, some progress has been made in sexual mating and pathogenic mechanism of this fungus, especially in molecular mechanism (Cai et al., 2022; Li et al., 2021a; Wang et al., 2019; Zhang et al., 2024; Zhu et al., 2019; Sun et al., 2019). For example, Cai et al. (2022) knocked out the *SsCI80130* gene which encodes squalene monooxygenase, in *S. scitamineum*. The resulting mutant exhibited a marked reduction in growth rate and a significantly diminished sexual mating ability. Further studies indicated that *SsCI80130* influences sexual mating and pathogenicity by regulating the synthesis of small-molecule signaling compounds required for mating, including cAMP (cyclic adenosine monophosphate) and tryptophol. Li et al. (2021a) identified the cytochrome P450 sterol 14 alpha demethylase, which encodes the cytochrome P450 family in *S. scitamineum*. The research results showed that the gene knockout mutant exhibited a pronounced reduction in sexual mating, and subsequent pathogenicity assays revealed a significantly lower incidence of disease in sugarcane when inoculated with the mutant in combination. Wang et al. (2019) identified an AGC kinase gene *SsAgc1*, and found that the *SsAgc1* gene plays an important role in the mating/filamentation, pathogenicity and oxidative stress tolerance of *S. scitamineum*. They also found that the *SsAgc1* signaling pathway may regulate tryptophol metabolism required for sexual mating of *S. scitamineum*. Recently, Zhang reported that major facility super family sugar transporter protein *SsMFSST1* regulated *S. scitamineum* mating, pathogenicity and sugar transport/absorption (Zhang et al., 2024).

Monocarboxylate permeases play an essential role in mammalian metabolism. Monocarboxylic acids-such as lactic acid, pyruvate, and acetic acid-as well as the tricarboxylic acid citric acid, are critical for cellular metabolism and homeostasis and must be efficiently transported by these permeases. The monocarboxylate transporter family (MCT8, MCT10) is reported to be primarily found in mammals and is crucial for intercellular metabolic communication (Friesema et al., 2003; Visser et al., 2007). Monocarboxylate permeases also play a significant role in bacteria. Hosie et al. (2002) identified the monocarboxylate permease gene *MctP* in the *Rhizobium leguminosurum*, and its knockout mutant was unable to grow on culture medium with alanine as the sole carbon source, indicating that the *MctP* gene is essential for alanine transport. Furthermore, monocarboxylate permeases are important in the pathogenicity of plant-pathogenic fungi. A study found that insertional disruption of the monocarboxylate transporter gene *BcMctA* in *Botrytis cinerea* significantly reduced the fungus’s growth on culture media containing monocarboxylates (acetic acid or pyruvate) as the sole carbon source. Pathogenicity assays demonstrated that gene disruption also significantly reduced the virulence of *B. cinerea* on cucumber and tomato (Cui et al., 2015). Zhao et al. (2023) identified a monocarboxylate transporter gene *MCT1*, in *Colletotrichum gloeosporioide*. The study revealed that this gene transport lactate, gene knockout mutants exhibited severe defects in nutritional growth, pigment deposition, and conidiation. Pathogenicity assays further showed that the knockout mutant completely lost its ability to infect apple leaves. Taken together, these findings indicate that monocarboxylate permeases are critical for metabolite transport, growth, and pathogenicity in mammals, bacteria, and fungi. However, to date, there have been no reports on monocarboxylate permeases in *Sporisorium scitamineum*. Therefore, it is necessary to investigate the role and regulatory mechanisms of monocarboxylate permeases in *S. scitamineum* to expand our understanding of the pathogenic mechanisms of this fungus.

Based on transcriptome sequencing data of different pathogenic strains *Ss16* and *Ss47*, previously isolated in our laboratory (Wu et al., 2020), we identified the monocarboxylate permease gene *SsMP1* as being significantly upregulated in the highly pathogenic strain *Ss16*. BLAST analysis predicted that the protein encoded by this gene functions as a monocarboxylate permease. In this study, we aimed to use polyethylene glycol (PEG)-mediated protoplast transformation technology to generate *SsMP1* gene knockout and complementary mutants. Through analyses of gene expression, phenotypes, sexual mating, and pathogenicity of wild-type, knockout, and complementary strains, we sought to elucidate the biological function of *SsMP1* in *S. scitamineum* and reveal the molecular basis by which it contributes to sexual mating and pathogenicity.

## 2 Materials and methods

### 2.1 Characterization of the *SsMP1* gene sequence

Based on our lab’s prior transcriptome data for *S. scitamineum*, *SsMP1*, which encodes a monocarboxylate permease, displayed significant differential expression (*p* ≤ 0.05) between the highly virulent strain *Ss16* and the less virulent strain *Ss47* (Wu et al., 2020). The *SsMP1*-encoded protein was characterized *in silico*: its isoelectric point and molecular weight were estimated with the pI/MW tool.^1^ The coding sequence was converted into amino acid sequence using an online translation tool.^2^ Conserved domains were identified in the NCBl database using Protein BLAST.^3^ Subcellular localization was predicted using WoLF PSORT.^4^ Secondary structure was forecasted using PRABI-Doua, and the three-dimensional structure was modeled with SWISS-MODEL. A systematic phylogenetic analysis of the *SsMP1*-encoded protein was conducted in MEGA 7 using the neighbor-joining method (Kumar et al., 2016; Saitou and Nei, 1987).

### 2.2 Strains and growth conditions

#### 2.2.1 Experimental material

The wild-type haploid strains *Ss16*^+^ and *Ss16*^–^were isolated and identified in our laboratory, and they are stored in a −80°C freezer (Deng et al., 2018). The culture media used in this study include YePSA solid medium (yeast extract 1%, peptone 2%, sucrose 2%, agar 2%), YePS liquid medium (yeast extract 1%, peptone 2%, sucrose 2%), YePS-soft semi-solid medium (yeast extract 1%, peptone 2%, sucrose 2%, agar 0.7%) and MM medium (K_2_HPO_4_ 0.205%, KH_2_PO_4_ 0.145%, NH_4_NO_3_ 0.05%, (NH_4_)_2_SO_4_ 0.03%, FeSO_4_ 0.001%, CaCl_2_ 0.001%, Glucose 0.2%, Z-Buffer 0.5%, pH 7.0). Z-buffer: MgSO_4_ 3%, NaCI 1.5%, ZnSO_4_ 0.01%, CuSO_4_ 0.01%, H_3_BO_3_ 0.01%, MnSO_4_ 0.01%, Na_2_MoO_4_ 0.01%. These materials are all from Sangon biotech Co., Ltd. (Shanghai, China).

#### 2.2.2 Growth test

The following experiment setup included wild-type haploid sporidia (*Ss16*^+^ and *Ss16*^–^), a SsMP1 knockout mutant (*ΔSsMP1^+^* and *ΔSsMP1^–^*), and a corresponding complement (*COMMP1*^+^ and *COMMP1*^–^). They were grown in 100 mL of YePS liquid medium at 28°C with shaking at 200 rpm for 24 h. Afterward, cultures were diluted into fresh YePS medium to reach a density of 105 cells/mL and incubated under the same conditions for another 48 h. Growth was monitored every 6 h by measuring OD_600_ with a NanoDrop 2000C spectrophotometer (Thermo Fischer Scientific, Wilmington, DE, United States).

To observe spore morphology, we inoculated the wild-type, knockout mutant and complementary mutant into a 50 mL centrifuge tube containing 10 mL of YePS liquid medium, and cultivated them in a constant temperature shaker at 28°C and 200 rpm until the OD_600_ was 1.00; Then, the morphology of haploid spores was observed under a 40 × optical microscope.

Dilute the spore solution of overnight cultivated wild-type, knockout mutant and complementary mutant to an OD_600_ of 1.00, and dilute them 10 times and 100 times, respectively. Then, take 1 μL each and drop them onto YePSA plates. Incubate at 28°C for 48 h to observe the growth of haploid spore colonies.

For the sexual mating assay, equal volumes of opposite mating-type strains (wild-type, knockout, or complement) were combined and inoculated onto YePSA medium (control) or YePSA medium supplemented with 5 mM cAMP or 0.02 mM tryptophol. Incubation occurred at 28°C in darkness for 42 h, after which photographs were taken to document results (Cai et al., 2022; Li et al., 2021a).

Abiotic stress tolerance tests were conducted by supplementing YePSA medium with 100 μg/mL Congo red (CR), 50 μg/mL Sodium Dodecyl Sulfate (SDS), or 500 mM NaCl, respectively, and MM medium with the same stressors. Cultures were incubated at 28°C in darkness for 48 h, followed by photographic documentation (Cai et al., 2022; Li et al., 2021b). The above experiments were repeated three times.

### 2.3 Nucleic acid manipulation

Genomic DNA was extracted using the modified CTAB method (Shen et al., 2006). PCR amplification was performed using Phanta Max super-Fidelity DNA Polymerase (Vazyme, P505, Nanjing, China). DNA fragments were purified using the FastPure Gel DNA Extraction Mini Kit (Vazyme, DC301, Nanjing, China). Total RNA was extracted using TRIZOL (Vazyme, R401, Nanjing, China) based on the manufacturer’s instructions (Supplementary material), and cDNA was synthesized using HiScript III RT SuperMix (Vazyme, R323, Nanjing, China). The concentration and purity of nucleic acids were measured using NanoDrop ND-1000 (Thermo Fischer Scientific, Wilmington, DE, United States).

### 2.4 Construction of *SsMP1* gene knockout and complementary mutant

The knockout strategy for the *SsMP1* gene involves homologous recombination using polyethylene glycol-mediated protoplast transformation with double-stranded DNA fragments (Cai et al., 2022; Li et al., 2021b; Cai et al., 2020). Two flanking fragments of the *SsMP1* locus were amplified from genomic DNA. In addition, overlapping fragments spanning the upstream and downstream regions were amplified using the hygromycin resistance gene (*hygromycin phosphotransferase, Hpt*). The upstream and downstream sequences were then fused separately to the corresponding ends of the *Hpt*gene, creating two fusion constructs. These fusion fragments were employed for protoplast transformation to generate *SsMP1* knockout mutants (40% PEG, incubate for 15 min). Following protoplast transformation, positive transformants were identified for the knockout mutant. After waiting for selective regeneration on the plate and appearance of transformants (Supplementary Figure 1), several transformants were picked aseptically with sterile tips in a biosafety cabinet and transferred into YePS liquid medium containing hygromycin (final concentration 100 μg/mL). Genomic DNA was extracted from the cultures using the CTAB method, and successful knockouts of the *SsMP1* gene were confirmed by PCR amplification. Supplementary Figure 2 presents a scheme of the knockout strategy.

The complementation of the *SsMP1* gene also employed polyethylene glycol-mediated protoplast transformation (Cai et al., 2022). The difference is that the complementary mutant not only replaces the *Hpt* gene with the target gene in the knockout mutant, but also carries the selectable *Zeocin* resistance gene. Supplementary Figure 3 presents a schematic diagram of gene complementation and the detailed process of supplementation. The primer design was based on the NCBI *S. scitamineum* genome sequence LK056692.1. Supplementary Table 1 lists all the primers related to the construction and validation of the knockout mutants and complementary mutant. The key steps and reagents involved in the gene knockout and complementation experiments are listed in Supplementary Table 2.

### 2.5 Analysis of gene expression

In order to evaluate the effect of *SsMP1* gene on the small molecule signaling substance cAMP and tryptophol synthesis required for sexual mating in *S. scitamineum*, using Quantitative real-time (qRT)-PCR to determine the expression levels of the cAMP synthesis key gene *Uac1* (encoding adenylyl cyclase) and the tryptophol synthesis key gene *Aro8* (encoding tryptophol synthase) (Chen et al., 2004). The study included three mating pairings: *Ss16*^+^ with *Ss16*^–^, *ΔSsMP1^+^* with *ΔSsMP1^–^*, *COMMP1*^+^ with *COMMP1*^–^. For each pairing, cultures were maintained at 28°C for 2 days, and total RNA was extracted with cDNA synthesized at 12-h intervals. qRT-PCR was then conducted on the ABI7500 Real time Fluorescent Quantitative PCR System (Thermo Fischer Scientific, Wilmington, DE, United States) using the ChamQTM Universal SYBR qPCR Master Mix (Vazyme, Q711, Nanjing, China) to quantify the transcripts of *Uac1* and *Aro8*. The amplification efficiency of qRT-PCR was from 94.8 to 101.8% (Supplementary Table 3). *ACTIN* served as the internal reference gene, and relative expression levels were calculated using the 2^–ΔΔ*Ct*^ method (Livak and Schmittgen, 2001). Three biological repeats each containing three technical replicates for each sample were performed.

Using the same approach as described above, we assessed *SsMP1* expression under three conditions: haploid sporidia growth, sexual mating, and the sugarcane bud infection process. Expression levels of *SsMP1* were tracked at 12-h intervals over a 72-h time frame.

The gene *SsMP1* expression levels of wild-type, knockout mutant and complementary mutant during the cultivation process in MM medium with citric acid as the sole carbon source were detected using the same method as above. During the 48 h cultivation process, gene expression levels were detected every 12 h. Three biological repeats each containing three technical replicates for each sample were performed. The primer sequences used are provided in Supplementary Table 1.

### 2.6 Evaluation of monocarboxylic acid transport capacity

For the experiment evaluating the transport capacity for both monocarboxylic and tricarboxylic acids, 0.2% citric acid, 0.2% acetic acid, 0.2% pyruvate and 0.2% lactic acid were added as the sole carbon source in MM medium without adding glucose. Cultivate the wild-type haploid sporidia, knockout mutant and complementary mutant of *S. scitamineum* on MM medium to OD_600 _ = 0.8, then dilute 10 times and 100 times, and take 1 μL each on the aforementioned MM plates for cultivation. During 48 h, 28°C, and dark cultivation, observe and take photos at 24, 36 and 48 h, respectively (Hosie et al., 2002).

### 2.7 Determination of citric acid concentration

Cultivate haploid sporidia (*Ss16*^+^, *Ss16*^–^, *ΔSsMP1^+^*, *ΔSsMP1^–^*, *COMMP1*^+^, *COMMP1*^–^) separately in MM medium with citric acid as the sole carbon source for 48 h (28°C, 200 rpm), during which the citric acid concentration in MM liquid medium is measured every 12 h. The concentration of citric acid is detected using a citric acid (CA) content detection kit (Sangon Biotech. Co., Ltd., Shanghai, China). The specific testing method is based on the operation manual (The main steps include sample centrifugation, filtration, and preparation of citric acid concentration standard curve).

### 2.8 Assay of the pathogenicity of the *SsMP1* gene knockout mutants and complementary mutants

The study utilized three genetic backgrounds of *S. scitamineum*: wild-type (*Ss16*^+^, *Ss16*^–^), a knockout mutant (*ΔSsMP1^+^*, *ΔSsMP1^–^*), and a complementary mutant (*COMMP1*^+^, *COMMP1*^–^). Each background was cultured in 10 mL of YePS liquid medium at 28°C with shaking at 200 rpm for 1 day. Spores were then collected by centrifugation, washed twice with ddH_2_O, resuspended in fresh YePS medium, and adjusted to a final concentration of 2 × 109 sporidia/mL. Equal volumes of sporidia from opposite mating types were mixed, and 200 μL of each mixture was injected into the stem growth point of the susceptible sugarcane cultivar “ROC22” (4–5 leaf stage). Seven mating-type combinations were tested: *Ss16*^+^ + *Ss16*^–^, *Ss16*^+^ + *COMMP1*^–^, *Ss16*^–^ + *COMMP1*^+^, *COMMP1*^+^ + *COMMP1*^–^, *ΔSsMP1^+^* + *ΔSsMP1^–^*, *Ss16*^+^ + *ΔSsMP1^–^*, and *Ss16*^–^ + *ΔSsMP1^+^*. Each combination included 48 plants (3 replicates, 16 plants per replicate). The *Ss16*^+^, *Ss16*^–^ pair served as the positive control, while sterile water inoculation served as the negative control. Inoculated plants were maintained in a greenhouse (temperature: 28–30°C, relative humidity: 60–80%, light: 11–13 h/day) for 5 months (the incubation period of sugarcane smut is long, and it takes 5–6 months from infection to full incidence under normal conditions), with sugarcane smut incidence assessed and recorded weekly. Diseased plants were marked during each survey to avoid re-counting, and infected whips were enclosed in plastic bags to prevent sporidia dissemination. Finally, the number of diseased plants and the incidence rate were calculated (Cai et al., 2022; Li et al., 2021b).

### 2.9 Microscopy

Images were captured with an Axio Observer Z1 microscope (Zeiss, Jena, Germany) fitted with a PCO Edge sCMOS camera (Kelheim, Germany).

### 2.10 Statistic analysis

Data were expressed as the means ± standard error (SE). One-way analysis of variance (ANOVA) with Duncan’s new multiple range test was used to analyze the significance of the differences among different treatments. Means were thought to be significantly different when *P* < 0.05. SPSS version 21 software (IBM Corp., Armonk, NY, United States) was used for one-way ANOVA.

## 3 Results

### 3.1 Identification and characterization of the *SsMP1* gene

**FIGURE 1:**
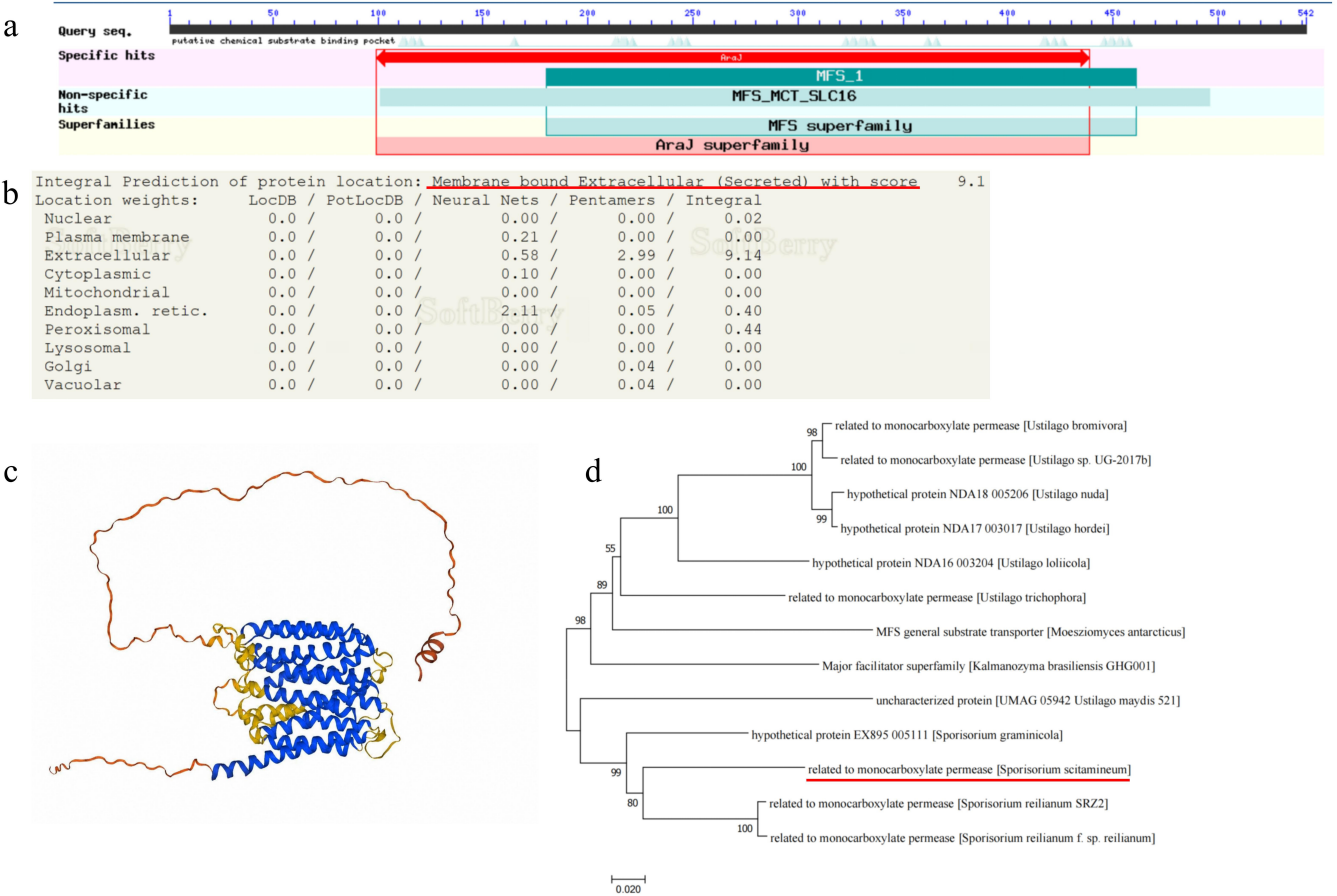
The structural domain and phylogenetic analysis of the *SsMP1* gene encoding the monocarboxylate permease in the *S. scitamineum*. **(a)** The predicted protein encoded by the *SsMP1* gene is the monocarboxylate permease. **(b)** Subcellular localization prediction of the protein encoded by the *SsMP1* gene. **(c)** The prediction of the tertiary structures of the protein encoded by the *SsMP1* gene. Different colors represent the consistency of the protein sequence. Blue indicates consistency > 80%, purple represents consistency between 70 and 80% and yellow indicates consistency < 50%. **(d)** Phylogenetic tree analysis of the protein encoded by the *SsMP1* gene, with Arabic numerals on the tree nodes indicating the confidence level of the phylogenetic tree (using the bootstrap method with 1,000 replicates). The protein is highlighted with a red horizontal line.

Based on previous transcriptome sequencing data in the laboratory, the gene *SsMP1* was identified as significantly (*P* ≤ 0.05) differentially expressed between the *S. scitamineum* strains *Ss16* (high pathogenicity) and *Ss47* (low pathogenicity). The gene spans 1,629 bp. According to NCBI protein sequence alignment, *SSsMP1* putatively encodes a monocarboxylate permease (NCBI protein accession CDU25901.1) comprising 542 amino acids. The predicted isoelectric point is 4.93 and the protein’s molecular weight is estimated at 58.5 kDa (Figure 1a). The subcellular localization predictions made using various tools consistently indicated that it might be located on the membrane bound Extracellular (Figure 1b). The predicted secondary structure comprises approximately 35.61% α-helix, 15.13% extended strand, 5.35% β-turn, and 43.91% random coil. The predicted tertiary structure conformation of the protein is shown in Figure 1c. *SsMP1* was predicted to have 12 transmembrane helices (Supplementary Figure 4). This study used SWISS-MODEL to predict the tertiary structure of *SsMP1* and integrated TMHMM to predict its membrane topology. Predictions for membrane-embedded regions often carry high uncertainty, and the accuracy of membrane boundaries and transmembrane segments may be limited. Phylogenetic analysis demonstrates homology of this protein with *Sporisorium reilianum SRZ2* conserved monocarboxylate permease and *Sporisorium reilianum f.* sp. *reilianum* conserved monocarboxylate permease, this indicates that the monocarboxylate permease encoded by the *SsMP1* gene is highly conserved in *Sporisorium* (Figure 1d). Additionally, conserved domain analysis via NCBI revealed that the *SsMP1* gene shared conserved domains of the MFS family with corresponding genes from *Botrytis cinerea* and *Colletotrichum asianum*, indicating that MFS transporters are conserved among these pathogenic fungi. Nevertheless, the presence of this domain in multiple pathogens that employ distinct infection strategies raises the hypothesis that it may contribute to fungal adaptation to the host environment. Further experimental validation is required to test its role in virulence (Supplementary Figure 5). This finding also suggests that *SsMP1* may function as a virulence factor, similar to the MFS transporters in these pathogenic fungi.

### 3.2 Molecular construction of *SsMP1* knockout mutants and complementary mutant

**FIGURE 2:**
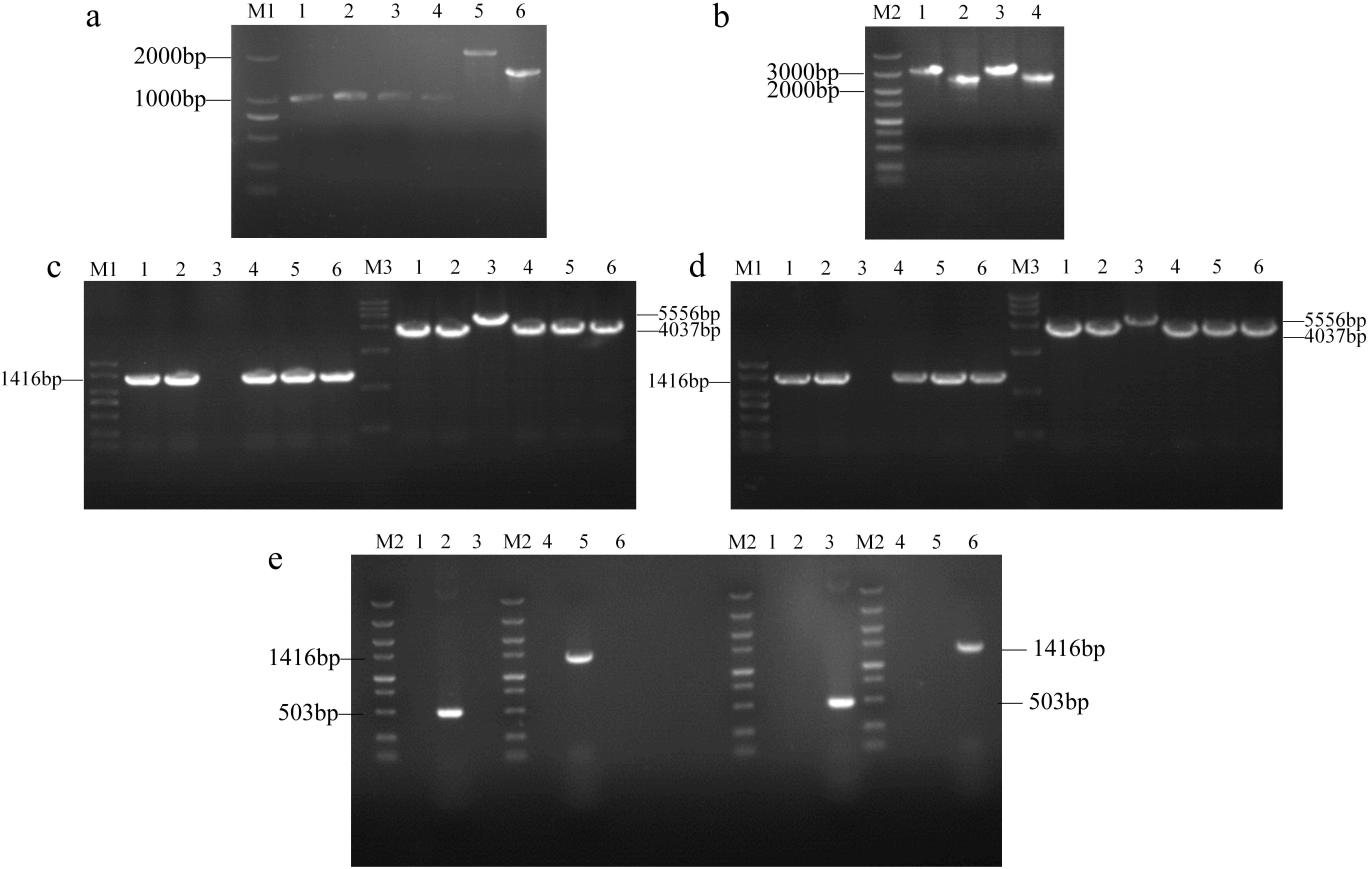
Construction and validation of *SsMP1* gene knockout and complementary mutant. M1 is 2,000 markers, M2 is 5,000 markers and M3 is 15,000 markers. **(a)** PCR amplification. Lanes 1, 3, and 2, 4 represent the left (LB) and right (RB) homologous arm fragments of the *SsMP1* gene in the wild-type *Ss16*^+^ and *Ss16*^–^ backgrounds, respectively. Lanes 5 and 6 represent two overlapping hygromycin fragments amplified by primers on Hpt-LB-F/HPT-LB-R and Hpt-RB-F/HPT-RB-R, namely the Hpt-up and Hpt-down fragments, with lengths of approximately 2 and 1.5 Kb. **(b)** Fusion PCR. Lanes 1 and 3 represent the fusion fragments of left homologous arm fragments and left hygromycin fragments in two wild-type backgrounds, namely the LB-Hpt-up fragments, while lanes 2 and 4 represent the fusion fragments of right homologous arm fragments and right hygromycin fragments in two wild-type backgrounds, namely the RB-Hpt-down fragments. **(c)** PCR validation of knocking out mutant *ΔSsMP1^+^*positive transformants in the *Ss16*^+^background. In Figure C, lanes 1, 2, 4, 5, and 6 represent the wild type, and lane 3 represents the transformant. The C diagram shows that lane 3 is a knockout mutant, and internal primers cannot amplify a 1,416 bp band for *SsMP1*-IN-F/*SsMP1*-IN-R. At the same time, due to the insertion of the hygromycin gene, external primers can amplify a 5,556 bp band for *SsMP1*-OU-F/*SsMP1*-OU-R. **(d)** PCR validation of knocking out mutant *ΔSsMP1^–^* positive transformants in the *Ss16*^–^ background. Lanes 1, 2, 4, 5, and 6 represent the wild type, while lane 3 represents the transformant. Similarly, lane 3 represents the knockout mutant. **(e)** Positive transformant PCR validation of complement mutants (*COMMP1*^+^and *COMMP1*^–^). The left half of **(e)** PCR validation of *COMMP1*^+^, with lanes (1, 4) and (3, 6) representing knockout mutants *ΔSsMP1^+^*. Lanes (2, 5) represent the transformants in the background of *ΔSsMP1^+^*. Lanes (2, 5) represent the complementary mutant *COMMP1*^+^. Endogenous primers for *SsMP1*-IN-F/*SsMP1*-IN-R and bleomycin primers for Zeocin-F/Zeocin-R can amplify 1,416 and 503 bp bands, respectively. Similarly, the right half of lanes (3, 6) is a complementary mutant *COMMP1*^–^.

The construction methods of *SsMP1* knockout mutant and complementary mutant are described in the Materials and Methods section. Using wild-type genomic DNA as a template, use *SsMP1*-LB-F/R and *SsMP1*-RB-F/R as primers to amplify two flanking fragments with lengths of 1,002 and 983 bp, respectively. In the same way, use the *Hpt* gene as a template, Hpt-LB-F/R and Hpt-RB-F/R are primers that amplify upstream and downstream fragment, with lengths of approximately 2 and 1.5 kb, respectively (Figure 2a). Subsequently, use *SsMP1*-LB-F/Hpt-LB-R and Hpt-RB-F/*SsMP1*-RB-R as primer pairs to fuse the two flanking fragments with the upstream and downstream fragments of the *Hpt* gene, respectively. The length of the fusion fragments is approximately 3 and 2.5 kb, respectively (Figure 2b). These two fused fragments were used for the subsequent wild-type protoplast transformation. As we expected, we successfully obtained knockout mutant (*ΔSsMP1^+^*, *ΔSsMP1^–^*). We used the external primer pair*SsMP1*-OU-F/R to detect a 5,556 bp band in the knockout mutants, but only a 4,037 bp band in the wild-type (Figures 2c,d). The internal primer pair*SsMP1*-IN-F/R was able to detect a 1,416 bp band in the wild-type, but no band was detected in the knockout mutant (Figures 2c,d). Similarly, after the complementary mutant (*COMMP1*^+^, *COMMP1*^–^) was successfully obtained, the internal primer pair *SsMP1*-IN-F/R could detect a 1,416 bp band, and the primer pair *Zeoncin*-IN-F/R could detect a 503 bp band while in the knockout mutants no band were detected (Figure 2e). It should be noted that the copy number of the supplemented gene was not independently quantified and was only confirmed by PCR, which represents a minor technical limitation of this complementation strategy.

### 3.3 Effect of *SsMP1* gene on haploid spore morphology, colony morphology and growth of *S. scitamineum*

**FIGURE 3:**
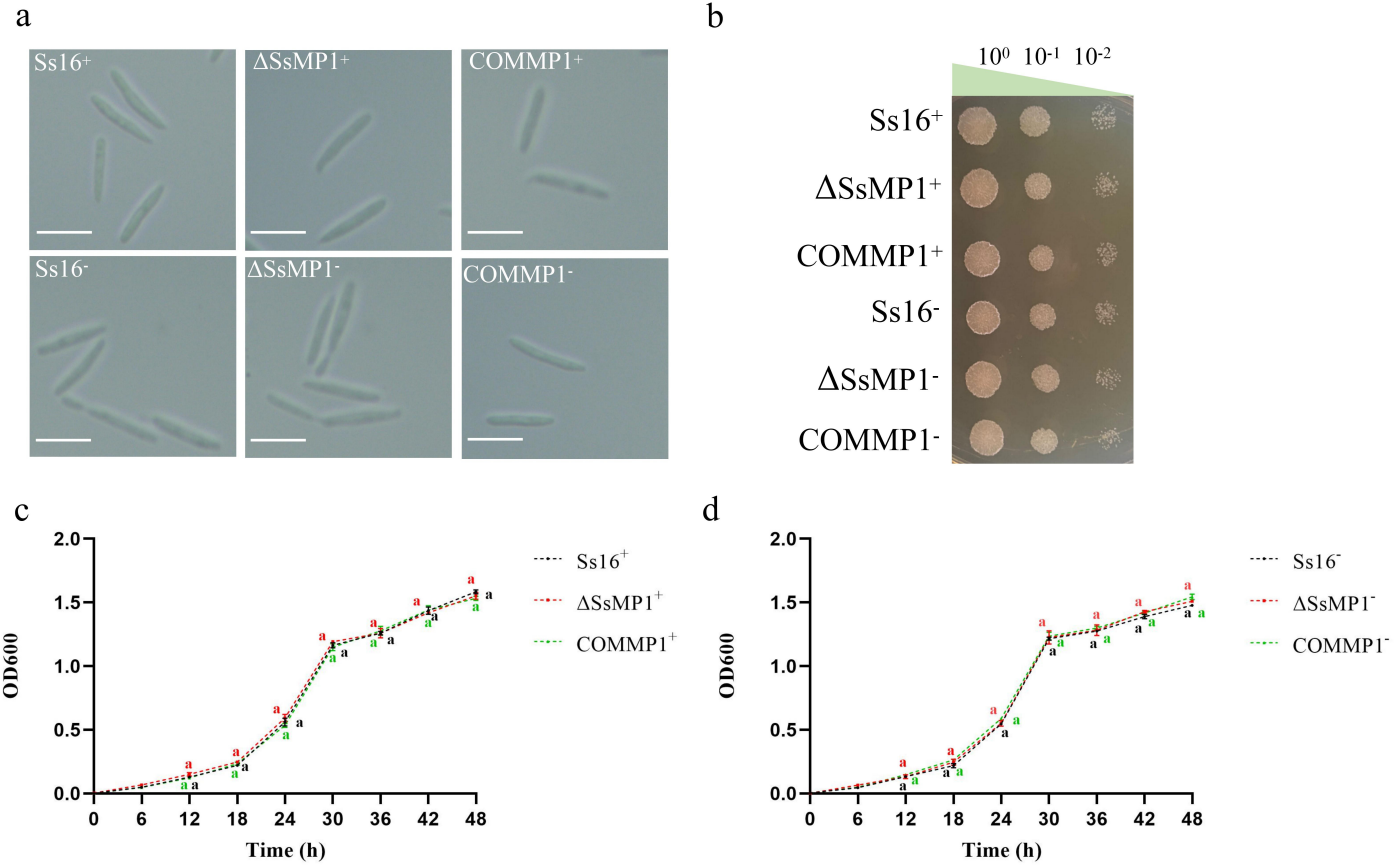
The effect of *SsMP1* gene on the morphology of haploid sporidia, colony morphology and growth curve of *S. scitamineum*. **(a)** The morphology of haploid sporidia from wild-type, knockout mutant and complementary mutant at 28°C for 48 h in YePS liquid medium (observed under a 40 × microscope), with a white bar representing 25 μm. **(b)** The haploid colony morphology of wild-type (*Ss16*^+^, *Ss16*^–^), knockout mutants (*ΔSsMP1^+^*, *ΔSsMP1^–^*), and complementary mutants (*COMMP1*^+^, *COMMP1*^–^) incubated on YePSA solid plates at 28°C for 48 h. **(c,d)** The growth curve of haploid sporidia, with three replicates per sample. The bar represents the standard error. The same lowercase letters indicate that there was no statistically significant difference at the 0.05 level (*P* < 0.05).

The microscopic observation results showed that the haploid sporidia morphology of the knockout mutant was the same as that of the wild-type and complementary mutant, both of which were elongated elliptical rods. This indicates that the *SsMP1* gene does not affect the haploid sporidia morphology of *S. scitamineum* (Figure 3a). The observation results showed that there was no significant difference in the morphology of haploid spore colonies among wild-type (*Ss16*^+^, *Ss16*^–^), knockout mutants (*ΔSsMP1^+^*, *ΔSsMP1^–^*), and complementary mutants (*COMMP1*^+^, *COMMP1*^–^) (Figure 3b). During the 48 h haploid sporidia culture process, OD_600_ values were detected every 6 h, and the results showed that the growth curves of haploid spores in wild-type, knockout mutant and complementary mutant were basically consistent (Figures 3c,d).

### 3.4 Effect of *SsMP1* gene on sexual compatibility of *S. scitamineum*

**FIGURE 4:**
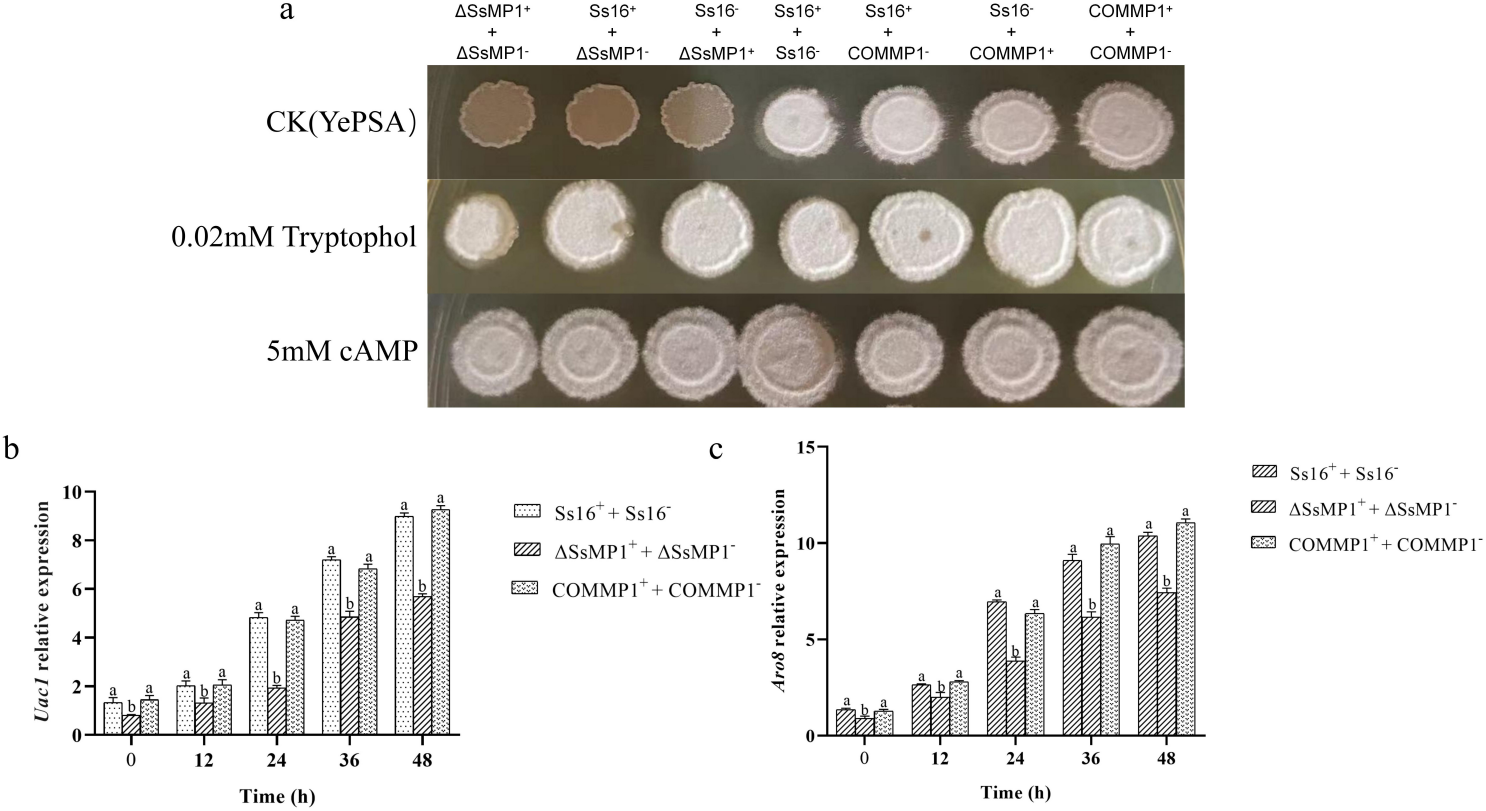
The effect of *SsMP1* gene on sexual mating of *S. scitamineum*. **(a)** The effect of adding cAMP (5 mM) or tryptophol (0.02 mM) to YePSA medium on the sexual mating ability of knockout mutants. Take photos at 42 h of cultivation, with untreated YePSA medium as the control. **(b,c)** The expression levels of *Aro8* and *Uac1* genes were measured during the 48 h sexual mating process of wild-type combination (*Ss16*^+^+*Ss16*^–^), knockout mutant combination (*ΔSsMP1^+^*+*ΔSsMP1^–^*) and complementary mutant combination (*COMMP1*^+^+*COMMP1*^–^). The bar represents the standard error. Different lowercase letters represent a significant difference at the 0.05 level (*P* < 0.05).

Take 1 μL of haploid sporidia solution of different mating types with an OD_600_ of 1.0 and mix them, then drop them onto a YePSA plate and incubate at 28 °C for 36–48 h to observe the growth of mycelium. The results showed that the knockout mutant combination (*ΔSsMP1^+^*+*ΔSsMP1^–^*), wild-type and knockout mutant combination (*Ss16*^+^+*ΔSsMP1^–^*; *Ss16*^–^+*ΔSsMP1^+^*) did not produce white villous hyphae, while the wild-type combination (*Ss16*^+^+*Ss16*^–^), complementary mutant combination (*COMMP1*^+^+*COMMP1*^–^) and wild-type and complementary mutant combination (*Ss16*^+^+ *COMMP1*^–^; *Ss16*^–^+ *COMMP1*^+^) all grew a large amount of white villous hyphae, indicating sexual mating ability of the knockout mutant has been completely lost, and this gene is involved in the sexual mating of *S. scitamineum* (Figure 4a). Adding an appropriate amount of exogenous small molecule signaling substances tryptophol or cAMP to YePSA medium, the knockout mutant combination (*ΔSsMP1^+^*+*ΔSsMP1^–^*), and the knockout mutant and wild-type combination (*Ss16*^+^+*ΔSsMP1^–^*; *Ss16*^–^+*ΔSsMP1^+^*) grew white villous hyphae (Figure 4a), indicating that the small molecule signaling substances tryptophol or cAMP can restore the sexual mating ability of the knockout mutant, indicating that the gene may be involved in the synthesis and transportation of small molecule signaling substances tryptophol and cAMP. During the 48 h sexual mating culture process, the expression level of the key gene *Uac1* for cAMP synthesis increased with the increase of culture time (Figure 4b). Among them, the gene expression level of the knockout mutant combination *Uac1* was significantly lower than that of the wild-type combination and the complementary mutant combination. The expression level of *Aro8*, a key gene for tryptophol synthesis, showed the same trend as *Uac1* (Figure 4c). Observing the morphology of mycelium under a 40 × microscope, it was found that the wild-type combination, the wild-type and knockout mutant combination and complementary mutant combination, there was almost no significant difference in the mycelium morphology (Supplementary Figure 6).

### 3.5 Determination of gene expression level of *SsMP1*

**FIGURE 5:**
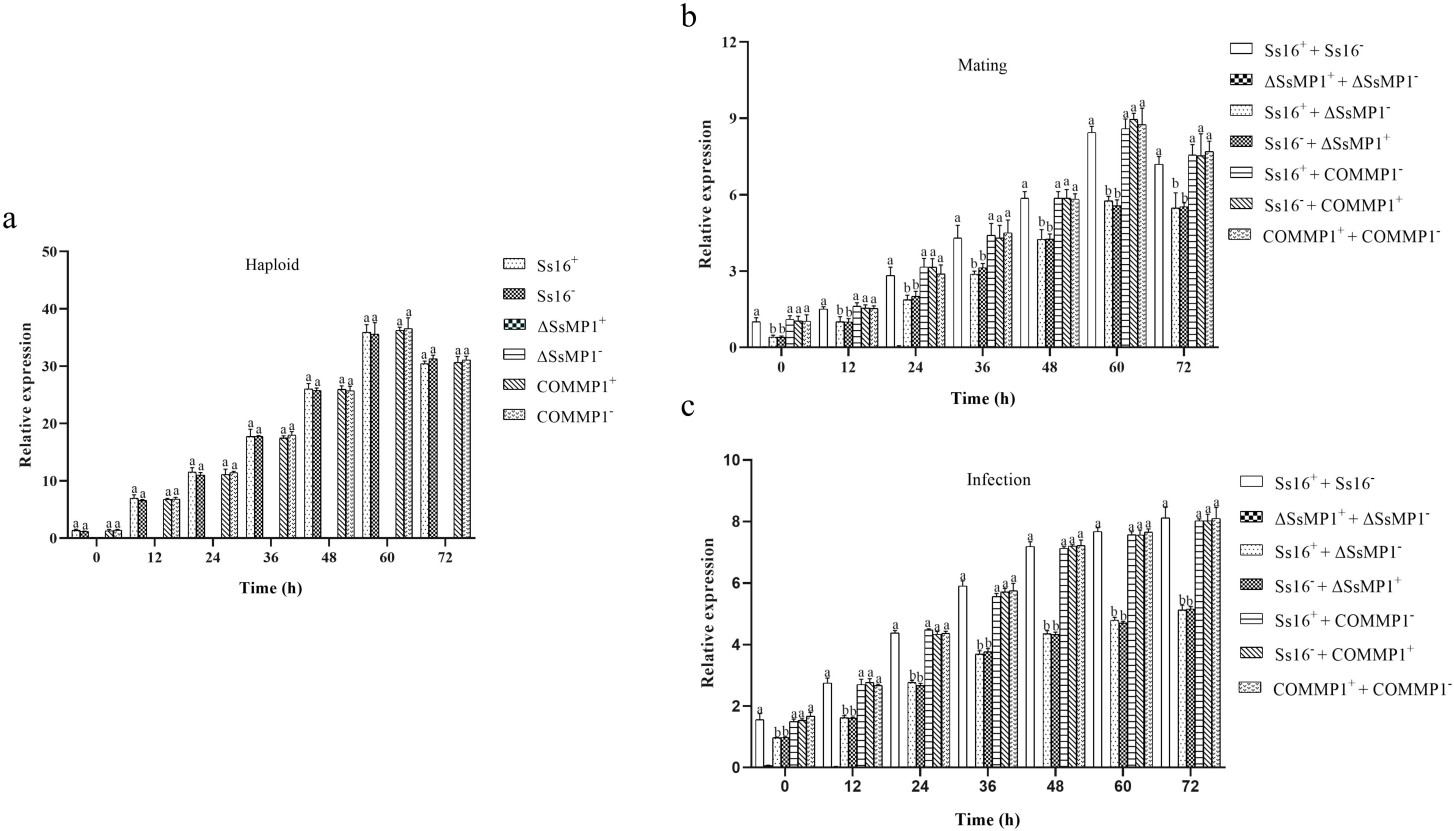
The expression level of *SsMP1* gene. **(a)** Expression levels during haploid sporidia culture. **(b)** The expression level during the process of sexual mating cultivation. **(c)** Expression levels during the sugarcane bud infection process. The bar represents the standard error from three independent replicates in each group. Different lowercase letters represent a significant difference at the 0.05 level (*P* < 0.05).

During the cultivation of haploid sporidia, the gene expression levels of wild-type (*Ss16*^+^, *Ss16*^–^) and complementary mutant (*COMMP1*^+^, *COMMP1*^–^) increased continuously with the passage of cultivation time and eventually reached saturation, reaching a peak at 60 h; Mutants (*ΔSsMP1^+^*, *ΔSsMP1^–^*) did not detect gene expression of *SsMP1*; The expression of this gene in complementary mutant (*COMMP1*^+^, *COMMP1*^–^) has been restored to a level equivalent to that of the wild-type (*Ss16*^+^, *Ss16*^–^) (Figure 5a). In 7 different sexual mating combinations, except for the mutant combination (*ΔSsMP1^+^*+*ΔSsMP1^–^*) where no expression of the *SsMP1* gene was detected, the expression level of the gene in all other combinations increased continuously with the increase of culture time, reaching its peak at 60 h, and then slightly decreased. Among them, the expression level of the gene in the wild-type and mutant combination (*Ss16*^+^+*ΔSsMP1^–^*; *Ss16*^–^+*ΔSsMP1^+^*) was significantly lower than that in the combination without mutant (Figure 5b). During the process of sugarcane bud infection process, the expression pattern of *SsMP1* gene showed a similar trend to that of the gene during sexual mating culture (Figure 5c).

### 3.6 Effect of *SsMP1* gene on abiotic stress tolerance

**FIGURE 6:**
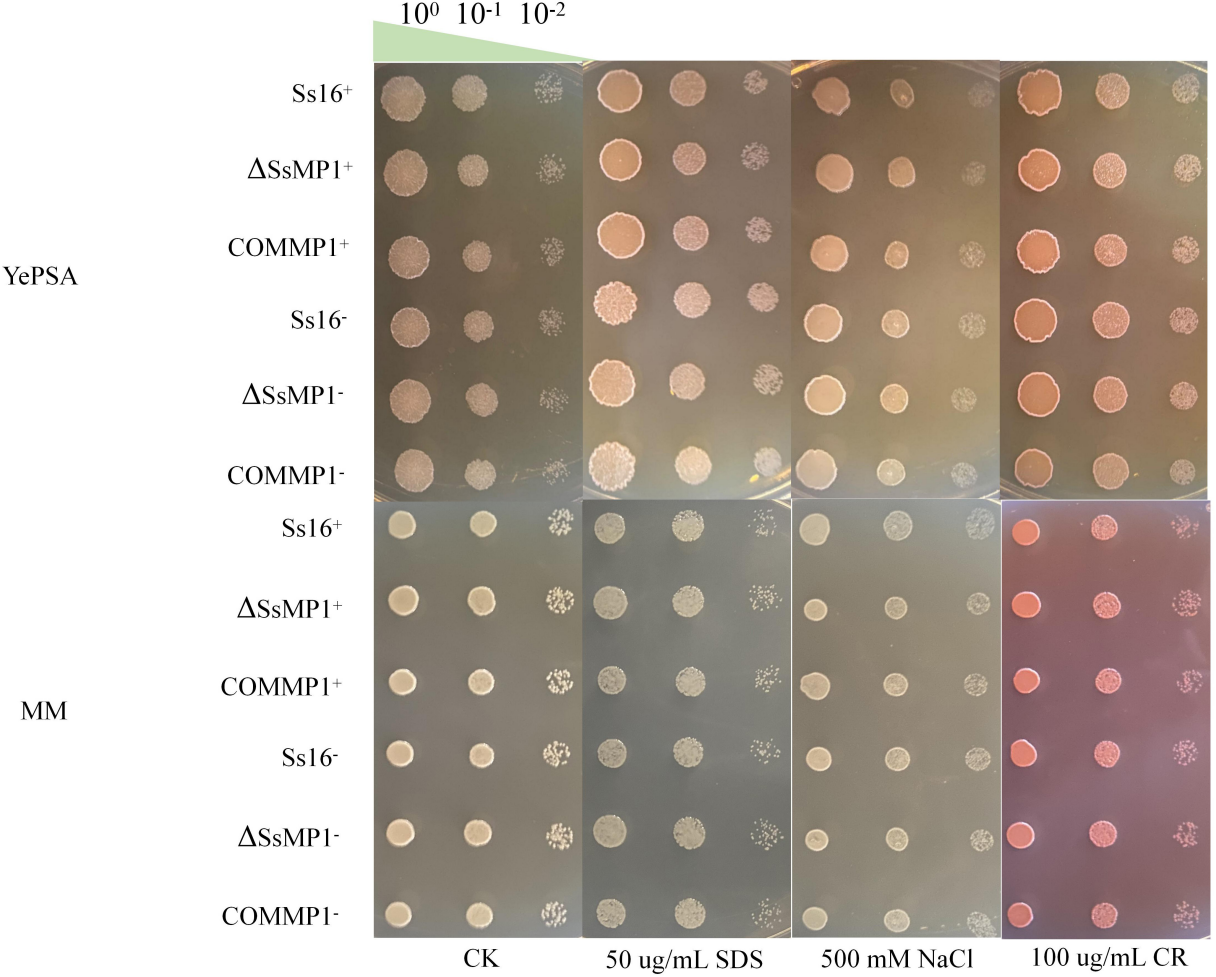
The effect of *SsMP1* gene on the abiotic stress tolerance of *S. scitamineum*. Inoculate continuously diluted wild-type (*Ss16*^+^, *Ss16*^–^), knockout mutant (*ΔSsMP1^+^*, *ΔSsMP1^–^*), and complementary mutant (*COMMP1*^+^, *COMMP1*^–^) onto YePSA and MM solid media supplemented with CR (100 μg/mL), NaCl (500 mM), and SDS (50 μg/mL), incubate at 28°C for 48 h, and using YePSA and MM solid media without exogenous abiotic stress substances as control.

We detected the tolerance of wild-type and mutant by adding abiotic stress substances such as SDS or CR (cell wall stress) and NaCl (hypertonic stress). The sporidia growth rates of wild-type (*Ss16*^+^, *Ss16*^–^), knockout mutant (*ΔSsMP1^+^*, *ΔSsMP1^–^*) and complementary mutant (*COMMP1*^+^, *COMMP1*^–^) were consistent on YePSA and MM media without any added abiotic stress substances. On YePSA and MM media with added concentrations of SDS, CR and NaCl, the sporidia growth rates of wild-type, knockout mutant and complementary mutant were consistent, indicating that the exogenous abiotic stress substances did not inhibit the growth of wild-type, knockout mutant and complementary mutant sporidia, indicating that the gene does not participate in oxidative stress responses under cell wall stress or hypertonic stress (Figure 6).

### 3.7 Effect of *SsMP1* gene on the transport and absorption capacity of monocarboxylic acid

**FIGURE 7:**
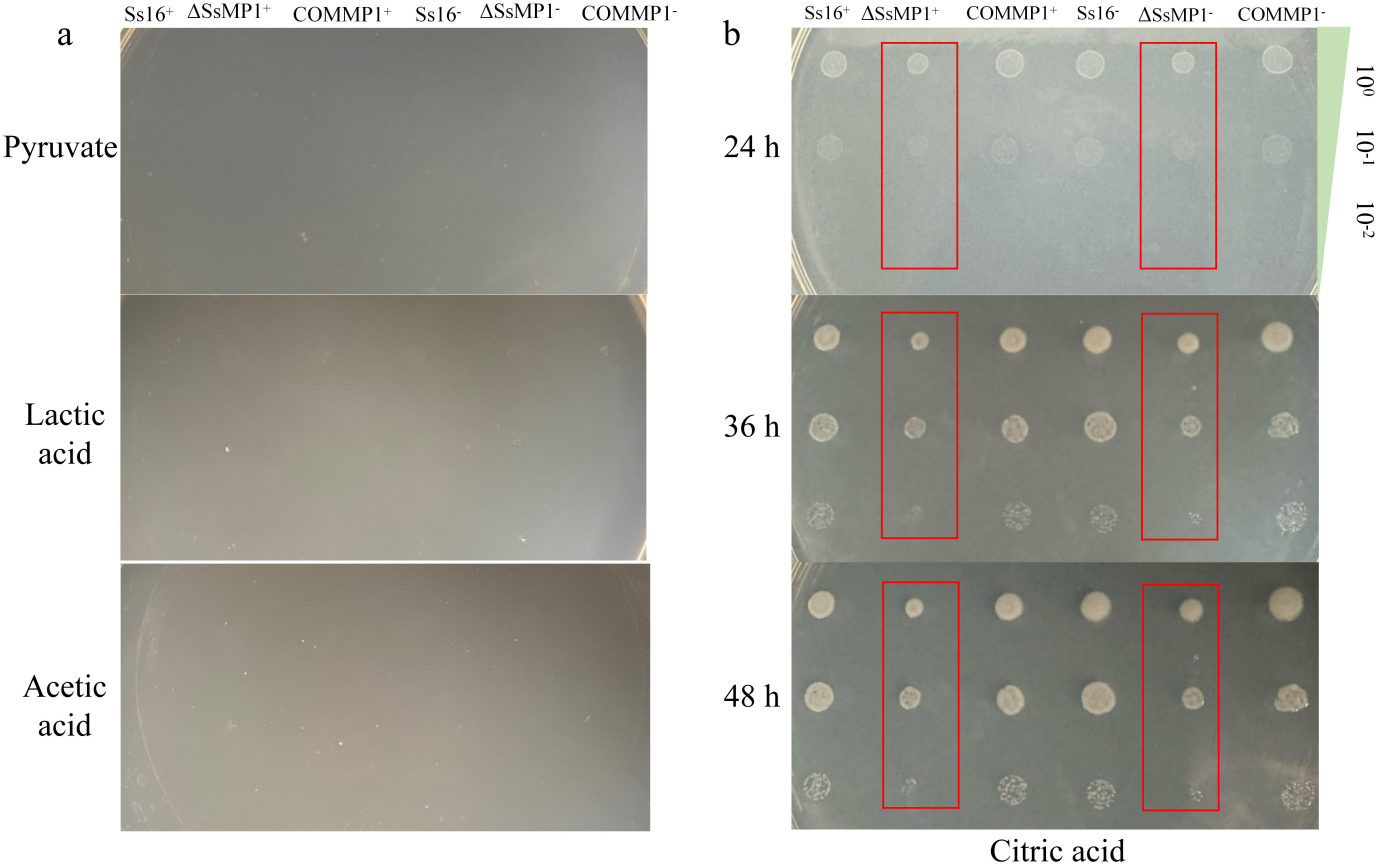
The effect of *SsMP1* gene on the ability of haploid sporidia of *S. scitamineum* to transport and absorb monocarboxylic acids. **(a,b)** Inoculate continuously diluted wild-type (*Ss16*^+^, *Ss16*^–^), knockout mutant (*ΔSsMP1^+^*, *ΔSsMP1^–^*) and complementary mutant (*COMMP1*^+^, *COMMP1*^–^) sporidia onto MM solid culture medium with pyruvate, lactic acid, acetic acid or citric acid as the sole carbon sources and incubate at 28 °C for 48 h.

Study the effect of gene *SsMP1* on the transport capacity for monocarboxylic acids (pyruvate, lactic acid, and acetic acid) and the tricarboxylic acid citric acid, using MM medium supplemented with each of these compounds as the sole carbon source. The research results found that the *S. scitamineum* cannot grow on MM medium containing pyruvate, lactic acid, or acetic acid as the sole carbon sources (Figure 7a); On MM medium containing citric acid as the sole carbon source, the growth rate of the knockout mutant was significantly slower than that of the wild-type strain and the complemented mutant over the time course of 24, 36, and 48 h, with the most pronounced difference observed at 48 h (Figure 7b), suggesting that *SsMP1* may participate in citric acid transport and uptake.

### 3.8 *SsMP1* gene is related to the transport and absorption of citric acid

**FIGURE 8:**
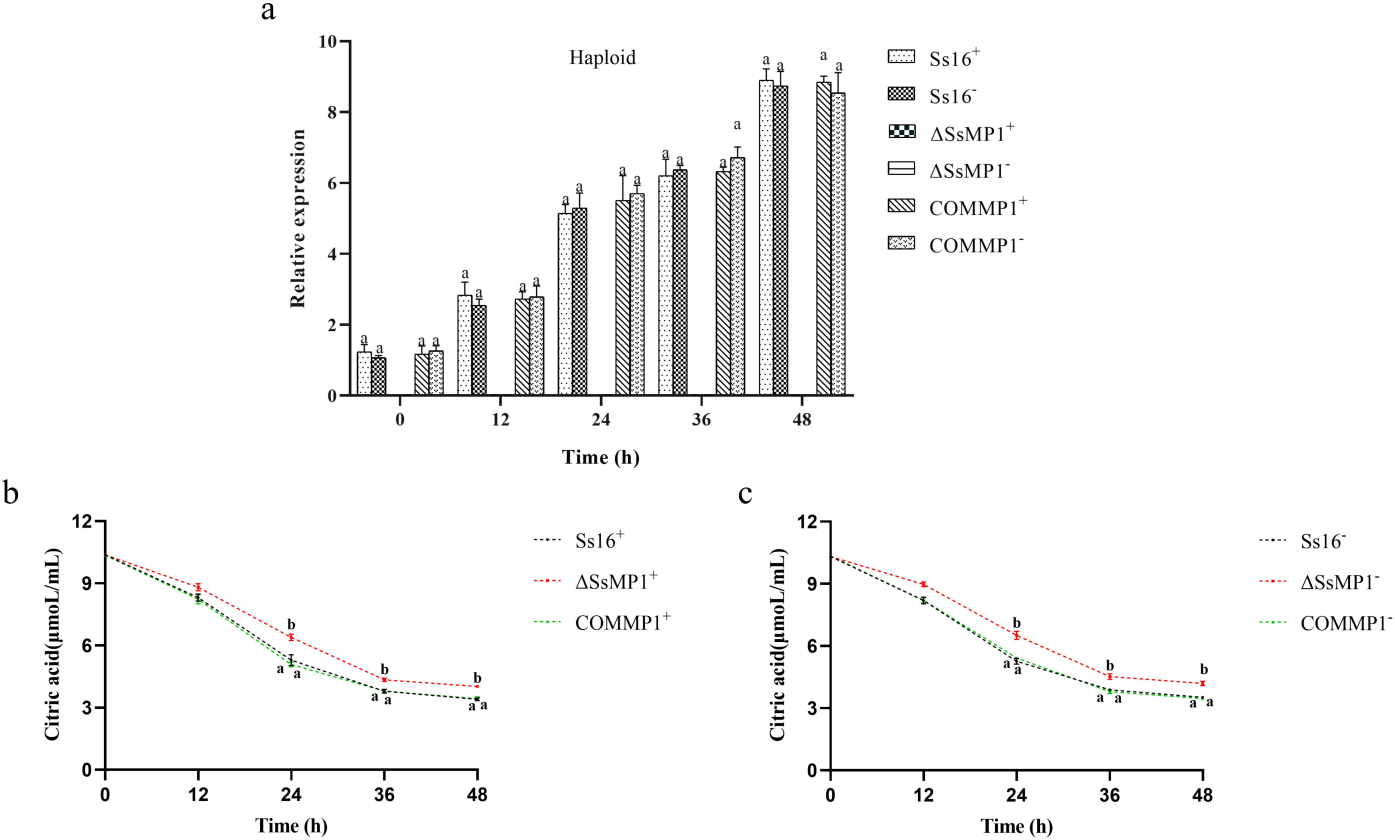
The *SsMP1* gene positively regulates the transport and absorption of citric acid by haploid sporidia of *S. scitamineum*. **(a)** The expression level of *SsMP1* gene was measured during the 48 h cultivation process at 28°C (in MM liquid medium with citric acid as the sole carbon source). **(b,c)** Changes in citric acid concentration in MM liquid culture medium with citric acid as the sole carbon source during 48 h of cultivation at 28 °C. The bar represents the standard error from three independent replicates in each group, and different lowercase letters represent a significant difference at the 0.05 level (*P* < 0.05).

To further verify the above results, wild type, knockout mutant and complementary mutant were cultured in MM medium with citric acid as the sole carbon source at 28 °C for 48 h in a constant temperature shaker. The expression level of *SsMP1* gene and the concentration of citric acid in the medium were measured every 12 h. The results showed that during the cultivation process, the expression of the gene was not detected in the knockout mutant, while the expression levels of the gene gradually increased with the increase of cultivation time in the wild-type and complementary mutant, and there was no significant difference in the expression levels of the gene between the wild-type and complementary mutant (Figure 8a). The concentration of citric acid in the culture medium for cultivating knockout mutant was significantly higher than that in the culture medium for cultivating wild-type or complementary mutant (Figures 8b,c). The above results further indicate that the indirect indication suggests that this gene may positively regulate the transport and absorption of citric acid by the haploid sporophytes of *S. scitamineum*.

### 3.9 Effect of *SsMP1* gene on pathogenicity of *S. scitamineum*

**FIGURE 9:**
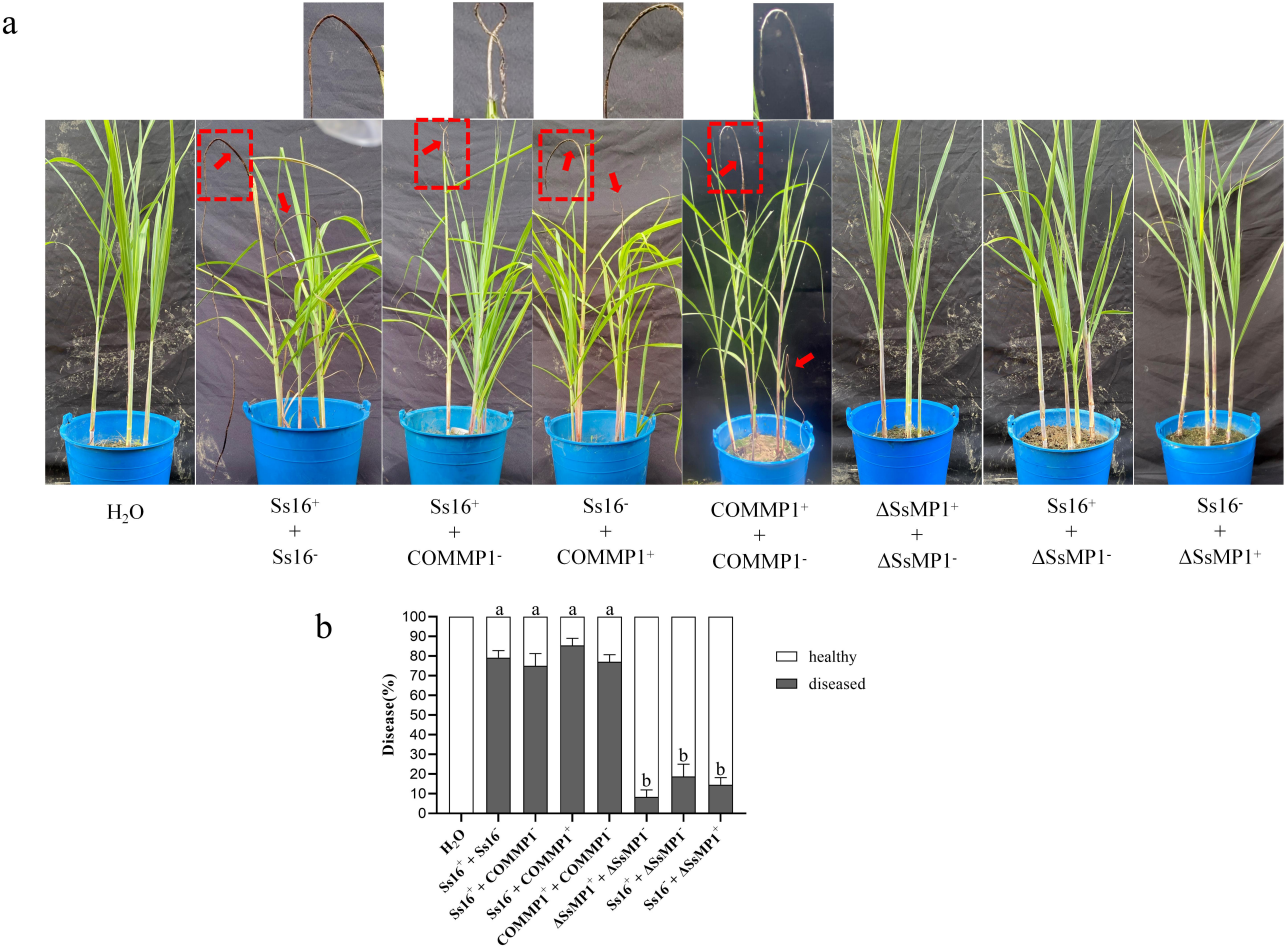
The *SsMP1* gene is involved in regulating the pathogenicity of *S. scitamineum*. **(a)** Symptoms of sugarcane smut disease. The red arrow points to the black whip, and the black whip in the red dashed box is magnified 20 times and located above the corresponding image. **(b)** The incidence rate of sugarcane smut in each inoculation combination. Sterile water is the negative control, and the wild-type combination (*Ss16*^+^+*Ss16*^–^) is the positive control. The bar represents the standard error from three independent replicates in each group, and different lowercase letters represent a significant difference at the 0.05 level (*P* < 0.05).

To identify the effect of *SsMP1* gene on the pathogenicity of *S. scitamineum*, we inoculated mixed sporidia suspension of opposite mating types to the susceptible variety “ROC22” of sugarcane smut disease. A total of 7 combinations were inoculated, including (*Ss16*^+^ + *Ss16*^–^), (*Ss16*^+^ + *COMMP1*^–^), (*Ss16*^–^ + *COMMP1*^+^), (*COMMP1*^+^+ *COMMP1*^–^), (*ΔSsMP1^+^* + *ΔSsMP1^–^*), (*Ss16*^+^ + *ΔSsMP1^–^*) and (*Ss16*^–^+ *ΔSsMP1^+^*). The wild-type combination served as the positive control, while sterile water inoculation acted as the negative control (Figure 9a). Inoculation with mixed sporidia lacking the knockout mutant (*Ss16*^+^ + *Ss16*^–^, *Ss16*^+^ + *COMMP1*^–^, *Ss16*^–^+ *COMMP1*^+^, *COMMP1*^+^+*COMMP1*^–^) produced high disease incidences of approximately 79.17, 77.08, 75.00, and 85.42%, respectively. By contrast, mixes that included knockout mutants (*ΔSsMP1^+^*+*ΔSsMP1^–^*, *Ss16*^+^+*ΔSsMP1^–^*, *Ss16*^–^+*ΔSsMP1^+^*) showed markedly lower incidences at 8.33, 18.75, and 14.58%, respectively (Figure 9b). In addition, we also found through tissue staining that the number of hyphae was extremely small or almost non-existent in the combination containing knockout mutants, while the number of hyphae was higher in the wild-type combination or the combination of wild-type and complementary mutant (Supplementary Figure 7). These results indicate that the *SsMP1* gene is involved in regulating the pathogenicity of *S. scitamineum*.

## 4 Discussion

This study was based on transcriptome sequencing data from two different pathogenic strains of *S. scitamineum*, *Ss16* and *Ss47*. Among the significantly differentially expressed genes, we identified a gene encoding a monocarboxylate permease, which we designated *SsMP1*. Using PEG-mediated protoplast transformation, we generated knockout mutants (*ΔSsMP1Δ* and *ΔSsMP1Δ*) and complemented mutants (*COMMP1*^+^ and *COMMP1*^–^). Phylogenetic analysis revealed that *SsMP1* is highly conserved in *S. scitamineum*. Importantly, in the knockout mutants, *SsMP1* expression was undetectable during haploid sporidia culture, sexual mating, or sugarcane bud infection, whereas expression in the complemented mutants was restored to wild-type levels. These results confirm that the knockout and complemented mutants constructed in this study are reliable and suitable for further functional analyses.

Monocarboxylate permeases are an important class of enzymes that play a crucial role in carbon metabolism and substance transport in fungi. On one hand, these enzymes participate in fungal energy metabolism and carbon source utilization, providing essential carbon and energy for growth and reproduction. For example, in *Saccharomyces cerevisiae*, the *ADY2p* gene has been identified as essential for acetate transport. Disruption of *ADY2p* significantly reduces acetate uptake, and the knockout mutant exhibits pronounced growth defects (Paiva et al., 2004). Similarly, Casal et al. (1999) identified a lactate transporter gene, *JEN1*, in *S. cerevisiae*. Disruption of *JEN1* prevented growth on a medium containing lactate as the sole carbon source, indicating a loss of lactate transport and uptake capacity. Therefore, monocarboxylate permeases are critical for the transport and absorption of substances in fungi. Our study yielded similar findings. We further investigated the transport and uptake capabilities of *SsMP1* toward monocarboxylic acids (pyruvate, lactic acid, and acetic acids) and a tricarboxylic acid (citric acid). When pyruvate, lactic acid, or acetic acid was provided as the sole carbon source in MM medium, *S. scitamineum* failed to grow, preventing assessment of the mutant’s ability to utilize monocarboxylic acids. This growth arrest may be attributed to defects in transport or the initial metabolic steps of these carbon sources, limiting their entry into the tricarboxylic acid (TCA) cycle. By contrast, citrate, as a tricarboxylic acid, might directly enter the TCA cycle through a relatively independent pathway, thereby bypassing this bottleneck (Underhill and Cabeen, 2022). These possibilities, however, require further experimental validation. In MM containing citric acid as the sole carbon source, the growth rate of the knockout mutants was significantly lower than that of the wild-type and complemented mutants, indicating specific defects in tricarboxylic acid utilization. There are many monocarboxylic acids, Only four monocarboxylic acids were tested in this study; therefore, whether citric acid is the only tricarboxylic acid substrate transported by *SsMP1* requires further investigation. Overall, our data primarily indicate that the mutant exhibits specific defects in the utilization of tricarboxylic acids such as citric acid. Among these, the monocarboxylic acid utilization pathway appears to be particularly sensitive to *SsMP1*-mediated regulation. Future substrate transport assays and transcriptomic analyses will help determine whether *SsMP1* functions as a direct transporter or as an indirect regulator. In summary, the growth arrest observed under pyruvate, lactic acid, and acetic acid conditions, in contrast to the normal growth supported by citric acid, provides a key functional contrast. This pattern suggests that the *SsMP1*-mediated regulatory network plays a critical role in distinguishing and prioritizing the utilization of tricarboxylic acid carbon sources. For example, in *Botrytis cinerea*, the absence of the monocarboxylate transporter gene *BcMctA* causes the knockout mutant to be sensitive to CR-containing medium, and the cell wall thickness is significantly reduced, reaching only about half of that in the wild-type, indicating that this gene is involved in the cell wall integrity stress response of *B. cinerea* (Cui et al., 2015). In the present study, three substances—SDS, CR, and NaCl—were added to the culture medium to assess the effect of the *SsMP1* knockout mutant on abiotic stress tolerance. The results showed no difference in colony growth between the *SsMP1* knockout mutant and the wild-type or complemented mutants, indicating that *SsMP1* is not involved in cell wall integrity maintenance or the response to hypertonic stress. This differs from the role of *BcMctA* in cell wall integrity stress response reported by Cui et al. (2015). This discrepancy may be related to the different types of monocarboxylic acids transported by each gene. In this study, *SsMP1* transports citric acid, whereas the *BcMctA* gene studied by Cui et al. (2015) transports pyruvate and acetic acid. Other possibilities also warrant further exploration. The mechanisms by which *SsMP1* participates in citric acid transport and related signal transduction (e.g., via redox state, metabolic flux, or intracellular pH) require further investigation. In addition, whether this gene regulates sexual mating and pathogenicity in *S. scitamineum* by participating in citric acid transport remains to be determined.

*S. scitamineum* is a typical dimorphic fungus, and only dikaryotic hyphae produced by the sexual mating of haploid sporidia with different mating types are pathogenic (Shen et al., 2013). The morphological transformation and pathogenicity of dimorphic fungi are highly dependent on the cAMP signaling pathway (Choi et al., 2015; Hogan and Sundstrom, 2009). In *S. scitamineum*, inhibition of cAMP synthesis impairs the sexual mating ability of haploid sporidia, preventing the formation of dikaryotic mycelium and, consequently, disease development. However, exogenous cAMP supplementation can restore sexual mating ability (Deng et al., 2018; Chang et al., 2019). *Ustilago maydis* is also a dimorphic fungus. In *U. maydis*, deletion of *Ubc1*, which encodes the cAMP subtype II regulatory subunit, disrupts morphological transformation and prevents normal sexual mating, thereby inhibiting dikaryotic mycelium formation (Klosterman et al., 2007). Similarly, in *Candida albicans*, cAMP modulates pathogenicity by regulating processes such as apoptosis, hyphal formation, and biofilm development (Kronstad et al., 1998). In addition, the exogenous small-molecule signaling compound tryptophol significantly influences sexual mating and pathogenicity in fungi, playing a key role in fungal biology. Exogenous addition of tryptophol can restore the sexual mating ability of an *SsAgc1* knockout mutant (encoding AGC kinase) in *S. scitamineum* (Wang et al., 2019). Therefore, cAMP and tryptophol serve as essential small-molecule signaling mediators for sexual mating in *S. scitamineum*. Our study produced similar findings. In experiments where the knockout mutant was treated with exogenous cAMP or tryptophol, the mutant regained sexual mating capability comparable to the wild type, accompanied by abundant production of white, villous hyphae. Whether the addition of exogenous signaling molecules alone can restore pathogenicity remains to be confirmed in future studies. Microscopic observations revealed no morphological differences between the recovered hyphae and those of the wild type. More importantly, qRT-PCR analysis showed that expression levels of *Aro8* and *Uac1* were significantly reduced in the knockout mutant compared to the wild type, whereas expression of the corresponding genes in the complemented mutant was restored to wild-type levels. Although transcript levels of *Uac1* and *Aro8* were altered, the absence of direct metabolite measurements (e.g., substrate/product concentrations or flux analysis) prevents the establishment of a direct causal link between transcriptional regulation and metabolic phenotypes. Future targeted metabolomics will be required to measure relevant metabolites to address this limitation. We did not systematically examine mating efficiency beyond 36ferehowever, because mating is essentially completed by 36ally examine mating efficiency beyond 36ferences between the recovered hyphae and those of the wild type. More tained *Uac1/Aro8* expression. Collectively, these findings suggest that *SsMP1* may regulate the production of small-molecule signaling substances essential for sexual mating in *S. scitamineum* by modulating key genes in the cAMP and tryptophol pathways, thereby influencing sexual mating and pathogenicity. However, the molecular mechanisms by which *SsMP1* regulates cAMP and tryptophol biosynthesis require further in-depth investigation. Future experiments, such as protein-protein or protein-DNA interaction assays, could be conducted to explore these regulatory mechanisms. In addition, it is necessary to compare cAMP and tryptophol levels among the knockout mutant, complemented mutant, and wild type to further validate whether the biosynthesis of these molecules is regulated by *SsMP1*. In summary, a novel pathogenic gene of *S. scitamineum*, the monocarboxylate transporter gene *SsMP1*, was identified in this study. Its pathogenic mechanism was preliminarily elucidated: *SsMP1* may affect the pathogenicity of *S. scitamineum* by regulating the expression of key genes involved in the synthesis of small-molecule signaling substances, such as cAMP or tryptophan, or by directly influencing their synthesis. Regarding the potential role of *SsMP1* in organic acid transport, growth phenotypes and citric acid depletion from the culture medium indirectly suggest that *SsMP1* may be involved in citric acid transport. However, direct evidence, including uptake assays using labeled citric acid, transport kinetics, or substrate specificity tests, is currently lacking. Therefore, these findings should be considered preliminary and hypothesis-generating rather than conclusive. This study enhances our understanding of the molecular mechanisms underlying *S. scitamineum* pathogenicity and may provide new targets for its prevention and control. Moreover, it expands knowledge regarding the biological functions of fungal monocarboxylate transporters, although the precise transport mechanism requires further validation. Future research should verify the subcellular localization of *SsMP1*, clarify its substrate specificity, and quantify cAMP and tryptophan levels to better define its role in signal transduction and virulence. Direct transport assays, such as radiolabeled or fluorescently labeled citric acid uptake, are also needed to conclusively establish the citric acid transport function of *SsMP1*.

## Data Availability

The datasets presented in this study can be found in online repositories. The names of the repository/repositories and accession number(s) can be found in the article/Supplementary material.

## Footnotes

1
https://web.expasy.org/compute_pi

2
https://www.novopro.cn/tools/translate.html

3
https://blast.ncbi.nlm.nih.gov/Blast.cgi?PROGRAM=blastp&PAGE_TYPE=BlastSearch&LINK_LOC=blasthome

4
https://www.genscript.com/wolf-psort.html
