# Supplementary material for: Monocarboxylate permease gene SsMP1 regulates Sporisorium scitamineum mating, pathogenicity and absorption and transport of citric acid
Source: Front Microbiol. 2026 Jul 8;17:1826158. doi: 10.3389/fmicb.2026.1826158 (PMC13388855; doi:10.3389/fmicb.2026.1826158)
Supplement: Supplementary file 1 [file Table_1.docx]

**Supplementary materials**

**Table S1.** Primers used in this study

| Name | Primer sequences (5’-3’) | Description |
| --- | --- | --- |
| *SsMP1*-LB-F^a^ | GCGATGCGGAACTCAAAGAC | Deletion |
| *SsMP1*-LB-R^a^ | GTCGTGACTGGGAAAACCCTGTATATAAGGCCAAGGGAAGGGG | construction |
| *SsMP1*-RB-F^a^ | GGTCATAGCTGTTTCCTGTGTGACTCGAATGTCACGCACACAC |  |
| *SsMP1*-RB-R^a^ | GGGCTGCATATGACTCGTCT |  |
| *Hpt*-LB-F^b^ | CAGGGTTTTCCCAGTCACGAC |  |
| *Hpt*-LB-R^b^ | GGTCAAGACCAATGCGGAGC |  |
| *Hpt*-RB-F^b^ | GCAAGACCTGCCTGAAACCG |  |
| *Hpt*-RB-R^b^ | TCACACAGGAAACAGCTATGACC |  |
| *SsMP1*-IN-F^a^ | CACCGAGTCAGATCGTCAATGATG | PCR verification |
| *SsMP1*-IN-R^a^ | ACGTCCCCGCAATAAACACT |  |
| *SsMP1*-OU-F^a^ | CTTCCGCGGCTATTTCTTGC |  |
| *SsMP1*-OU-R^a^ | GAAAGAGTGGCGGCAAAAGG |  |
| *SsMP1*COM-F^a^ | ATCCAAGCTCAAGCTAAGCTTCTCGATGCCGCAGTA  GGATT | Complementation  construction |
| *SsMP1*COM-R^a^ | CAGCAAGATCTAATCAAGCTTAGTCGACGAAAGGA  CACCAC |  |
| COM-HPT-LB-F^b^ | GCGCGCGTAATACGACTCAC |  |
| Zeocin-R^b^ | GAAGTGCACGCAGTTGCCG |  |
| Situ-F^b^ | CTCCGTGTTGATGCTGGGAC |  |
| COM-HPT-RB-R^b^ | CGAGCATTCACTAGGCAACCA |  |
| Zeocin-IN-F^b^ | CTGTGATCAGCAGCCAAT | PCR verification |
| Zeocin-IN-R^b^ | GTCAACTTGGCCATGGTG |  |
| *SsMP1*-qF^a^ | GCTCTCACCGAGTCAGATCG | qRT-PCR |
| *SsMP1*-qR^a^ | TGGTAGCGACGTCTTGATCG |  |
| *Aro8*-qF^b^ | CCTGGTGTTGCGTTCATTCC |  |
| *Aro8*-qR^b^ | CAAGCTCGGGCATCGTCTTA |  |
| *Uac1*-qF^b^ | CTGACGGAGATGTAGCCAAAG |  |
| *Uac1*-qR^b^ | AACGAGACAAGGAGGGAGTA |  |
| *Actin*-qF^b^ | ACAGGACGGCCTGGATAG |  |
| *Actin*-qR^b^ | TCACCAACTGGGACGACA |  |
| *SsMP1*-pCOM-F | CCGGTAGTATTTCGCACGGA |  |
| *SsMP1*-pCOM-R | GTCCCGGAAGTTCGTGGACA |  |

"a" indicates that the primer sequence was designed in this study, and "b" indicates that the primer sequence was sourced from reference [15].

**Table S2.** Key reagents involved in construction of *SsMP1*gene knockout and complementary mutant in this study

| Reagent | Source | Purpose |
| --- | --- | --- |
| PEG-3350 | sigma | Protoplast transformation |
| lysing enzymes | sigma |  |
| Heparin sodium | sigma |  |
| Sorbitol | sigma |  |
| Hygromycin,Hyg | Invitrogen | Resistance screening |
| Bleomycin,Zeocin | Invitrogen |  |

**Table S3.** Amplification efficiency of qRT-PCR in this study

| Gene | Amplification efficiency (%) | Slope of the standard curve | Linear correlation coefficient |
| --- | --- | --- | --- |
| *Actin* | 98.8 | -3.35 | 0.999 |
| *Uac1* | 97.6 | -3.38 | 0.998 |
| *Aro8* | 101.8 | -3.28 | 0.999 |
| *SsMP1* | 94.9 | -3.45 | 0.997 |

**
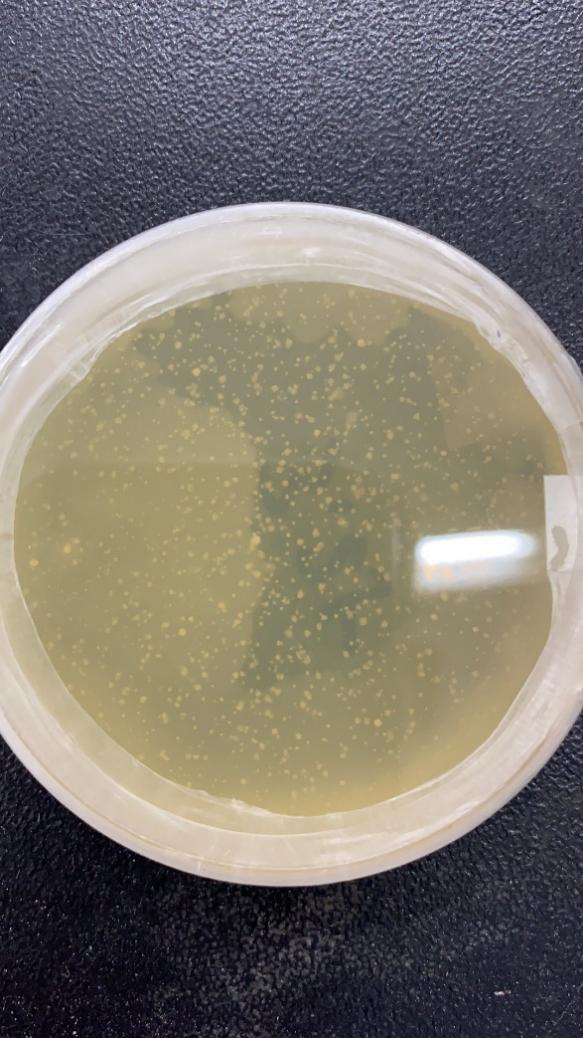
**

**Figure S1.** Selection plate for positive transformants of the SsMP1 knockout mutant

**
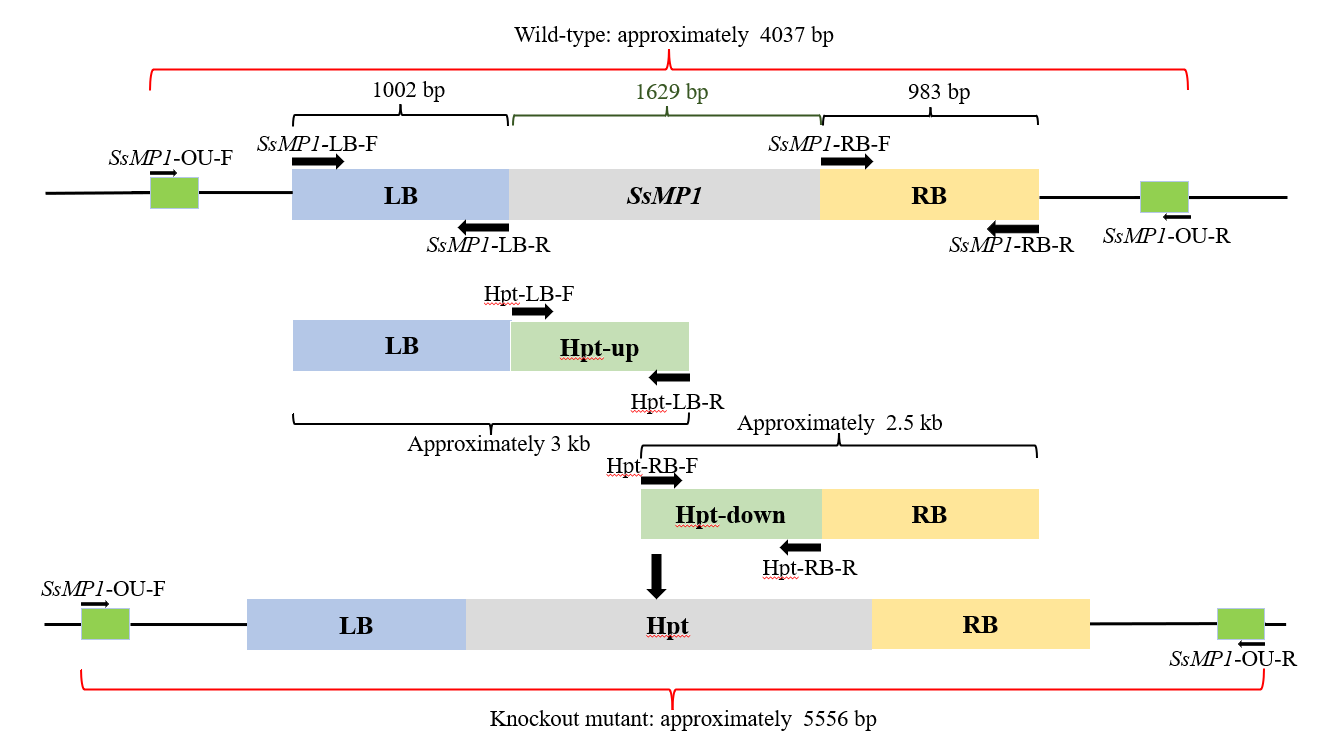
**

**Knockout sequencing results-***SsMP1*-OU-F/R

cttccgcggctatttcttgctcggcgttttcttcgggccaagttttcataaaacgaagaaatctgcgaatggaaaaaatggggaaaaaaaatgccggtctgctgcacagtctggctcgcgtggaaaagcgcagcagctgtcaaaaaaaaaaaaaaaagcgtttacccgataagcaagcctgattctcgccgtctcagctcaacttgaacgccagcaacagggcaatcgagctggcttctcctctactgttatcggctaaagacagccagccgagcttcgagtcatgctcgggttctggtctcccggtgaagcgacgttcgacctcaagctctgcgtctaggtttggtatgcgtgagattcccaacaacaagcgatgcggaactcaaagacttggcattgatgccaccgcggaggatgagcccgttcttccgggttcgatgcacagctgcatctttgatcaaaggcgcttcgacgtttaacgttgtgcggttgtcctgtcttgttggtcgctgttggaaaagcaaaattgcccggcgaacacgccgaacgtcgtttgcagcagcatcttgttctttgcggagacgagttggtgatttcatgcaaaaacctgcatgcttccatcgcagtcgcagcatttcgcgtgggctccaaggggcgctagaggaggatcagagggcggccagacgttctaagcagttgctgcaaagttgacagcggcttcgggtgaattttccgcctgaaatcgtcttgtctatcaaaccaaatttgatcctctcttgagctttctcccaacctctcgggatgggtcaaccttgtcccatccatcgttagcatgggcttcccacgaccactgtggatttgtgtttgaacatactcacgcccatccttcctccttcacacgcacacattttgacgcaaacagcgttgcatgaaccgtaccgttcggacaagccgtgactcgttggtccccaccaaaaacataaaccttaccagcgcgctgtctctgcgcggctgcctctgcagtccggcttcatctcggcatctttccgacgcaaaagcaaccctcttttcttgtacagtcacaacgatcgagggtggcaaagcgcagcagcagtgtcgtttgactcagcctgctgcgaagaagactgccgtagtcagcgctttgatttgaacaataaaagagagactgactcagcttgtccccaccgcagcagcccctctttctttctttcagtcaaatggcgacatctttctaacctcatcacgccatacactttgaccttcagtcgcacatctccgttacacgctaacaattgaccccccccccccccccttcccttggccttatataCAGGGTTTTCCCAGTCACGACGTTGTAAAACGACGGCCAGTGAGCGCGCGTAATACGACTCACTATAGGGCGAATTGGGTACCGGGCCCCCCCTCGAGCGATCCTTGAAGCTGTCCCTGATGGTCGTCATCTACCTGCCTGGACAGCATGGCCTGCAACGCGGGCATCCCGATGCCGCCGGAAGCGAGAAGAATCATAATGGGGAAGGCCATCCAGCCTCGCGTCGAGCTTTGAAGTTGCTGCAAGCTGGCTTCAAGCCATCCCATCCGAATGTGATGGATGCGTTCTTTCTGGGCCGTTGCGACTTTGGGGATCGTCTTTCCCGCGCCCTTGGTTGGAGGCCCTGTCTCCGGTGTCCCTTGTCCCTTCCAGGCAAGCGAGCGAGGTCCATTCAGATGGTGCTCCATCAGCGTTGGCTTTCCGTCCCCATTGGCTCTTGGCAATTCGGTCAGCGGGGCTGACTGCCTCAGGTGGGGCAGTGCTAGTGTGTGTACCGACCCGCAGGATTGGTGCTTTGCCCAGAGCTCTACAGAATAGCGCGCGCATCCATATGTTAGTTCTGCAATTTTCTTGTATCGGTGCTGTGACTCATACTTCCCCCTTTGGCTGGCCTTGCGGCAACCAATAAGAACGCACAGTGAAATCTTGCGGGTGGGGAGTGGATCCATGGCGCCTGCATTGGCTTGGGGACGCGCACTGTCGCACACTTCCATCTGACCTTTCAGAAGGGTTTCGTGGTGGGCAAGGACCAACCGGTTGCGCGGCGTGCGTGGGTGCCTCGCCCGGCACTGCCAGGGCCACTGCAGTGGCAGTTTGCTGCCTGATACAAAATCCTTCCCTCCGCCCAGTTTTCCCTCTTTGACCTTCCTTTCTCTTCTCTGCAACCAAATCCACCCTATCAAACCAAAACAGTATCTCGACCGAGGTATCAACCTGAATCAGCAACATCGTAGCCAGCATTTGTCTCCGTCTCTGCAGAACCAGCGAGTTGCAAACATTATCCAGGCAACAGGGCACCAACTCACTTCTTCGGCTTTCACCAATCGGTACAGCTCTTCTCAGAACTCGCGTCCGCAACAGTTCTACGCTTCCTCAGCACCTTCTTCAGCTTCAATCCTGAACACTCAGAACCGCGCACAGCAGCGCCCTCCTGTTCCCTTGTTTCCCAAAAGTACCGGTAGTATTTCGCACGGAAAGCAGGGCAACAAGATGTTCTCAGGTACCCATATGAAAAAGCCTGAACTCACCGCGACGTCTGTCGAGAAGTTTCTGATCGAAAAGTTCGACAGCGTCTCCGACCTGATGCAGCTCTCGGAGGGTGAAGAATCTCGTGCTTTCAGCTTCGATGTAGGAGGGCGTGGATATGTCCTGCGGGTAAATAGCTGCGCCGATGGTTTCTACAAAGATCGTTATGTTTATCGGCACTTTGCATCGGCCGCGCTCCCGATTCCGGAAGTGCTTGACATTGGGGAATTCAGCGAGAGCCTGACCTATTGCATCTCCCGCCGTGCACAGGGTGTCACGTTGCAAGACCTGCCTGAAACCGAACTGCCCGCTGTTCTGCAGCCGGTCGCGGAGGCCATGGATGCGATCGCTGCGGCCGATCTTAGCCAGACGAGCGGGTTCGGCCCATTCGGACCGCAAGGAATCGGTCAATACACTACATGGCGTGATTTCATATGCGCGATTGCTGATCCCCATGTGTATCACTGGCAAACTGTGATGGACGACACCGTCAGTGCGTCCGTCGCGCAGGCTCTCGATGAGCTGATGCTTTGGGCCGAGGACTGCCCCGAAGTCCGGCACCTCGTGCACGCGGATTTCGGCTCCAACAATGTCCTGACGGACAATGGCCGCATAACAGCGGTCATTGACTGGAGCGAGGCGATGTTCGGGGATTCCCAATACGAGGTCGCCAACATCTTCTTCTGGAGGCCGTGGTTGGCTTGTATGGAGCAGCAGACGCGCTACTTCGAGCGGAGGCATCCGGAGCTTGCAGGATCGCCGCGGCTCCGGGCGTATATGCTCCGCATTGGTCTTGACCAACTCTATCAGAGCTTGGTTGACGGCAATTTCGATGATGCAGCTTGGGCGCAGGGTCGATGCGACGCAATCGTCCGATCCGGAGCCGGGACTGTCGGGCGTACACAAATCGCCCGCAGAAGCGCGGCCGTCTGGACCGATGGCTGTGTAGAAGTACTCGCCGATAGTGGAAACCGACGCCCCAGCACTCGTCCGAGGGCAAAGGAATAGAGTAGATGCCGACCGGGATCCACTTAACGTTACTGAAATCATCAAACAGCTTGACGAATCTGGATATAAGATCGTTGGTGTCGATGTCAGCTCCGGAGTTGAGACAAATGGTGTTCAGGATCTCGATAAGATACGTTCATTTGTCCAAGCAGCAAAGAGTGCCTTCTAGTGATTTAATAGCTCCATGTCAACAAGAATAAAACGCGTTTCGGGTTTACCTCTTCCAGATACAGCTCATCTGCAATGCATTAATGCATTGGACCTCGCAACCCTAGTACGCCCTTCAGGCTCCGGCGAAGCAGAAGAATAGCTTAGCAGAGTCTATTTTCATTTTCGGGAGACGAGATCAAGCAGATCAACGGTCGTCAAGAGACCTACGAGACTGAGGAATCCGCTCTTGGCTCCACGCGACTATATATTTGTCTCTAATTGTACTTTGACATGCTCCTCTTCTTTACTCTGATAGCTTGACTATGAAAATTCCGTCACCAGCCCCTGGGTTCGCAAAGATAATTGCACTGTTTCTTCCTTGAACTCTCAAGCCTACAGGACACACATTCATCGTAGGTATAAACCTCGAAAATCATTCCTACTAAGATGGGTATACAATAGTAACCATGGTTGCCTAGTGAATGCTCGACGGTATCGATAAGCTTGATATCGAATTCCTGCAGCCCGGGGGATCCACTAGTTCTAGAGCGGCCGCCACCGCGGTGGAGCTCCAGCTTTTGTTCCCTTTAGTGAGGGTTAATTGCGCGCTTGGCGTAATCATGGTCATAGCTGTTTCCTGTGTGAggtgagactcgaatgtcacgcacacacgggggttcctgtgcaaaacgcatccacattccaacacattaaaactatacagaccgttctctctctactttttttcccgttggctggtgttcgtgaatcatatacggccattccacataccccttctccgccctctcgtcctcgaccctatctacaccaagcaccctaaacccagccttcaaataagcccccttccccatcgggctgctctccaaccacgcatccaccccttccttatccgccttcctcgtgccccacctgaccaacatcgtccccaaccccttcctctgaaatcccggatccaccaccaacagcttcaaaaaccaatacaccctcccctgcatcacaccttccctcgtccgattcatccttgcgaaaaacctgttcaacagcgtcggttcctcctccgctttcttatcgctttcctcgctgtctggtggtgtcctttcgtcgactttctgccagatgcccaacccaactagtgaagagccttcggggaggtcggaggagtctttatcttctcccaaaccgtcgttggtgatgtcggtgttggggggtaggaggtaggtgcctacgaggaagaggttgggtttagggtagagttgggaggtgagggttttgagtttggcgatggattcgagacgggtgagggggcgttggaggttggggtcgatttcgagggagaggaggtgggatagttcgtcaggggtggtgttgtgggggttggtgaagaaggcgtcgtgacggatgcgtgcgatggaggggacttcggaggggcgcgtgaggcgcgttacgaattgtggtgtgggttgagtcatgttgtatagttcaagaggggtggcggagggtggggaaggtgggaggagggggaggagggatgggggaggagaaggaggtatataccgagtgaagggaatgaaagacgagtcatatgcagccccggaaggtgatggtccgagccataatgttgctgtcaagcggggcgtggcagtgcggaaaattcgccgtaaccttttgccgccactctttc

**Figure S2.** Schematic diagram of gene knockout. The method for preparing protoplasts is as follows. The Ss16⁺ and Ss16⁻ strains were first cultured separately in YePS liquid medium overnight, then transferred to fresh YePS medium and adjusted to an OD₆₀₀ of 0.1–0.2. After further incubation for approximately 12 h until the OD₆₀₀ reached about 0.7, the cells were collected and washed successively with SCS (Sodium citrate 0.6% (w/v), sorbitol 18.2% (w/v), pH 5.8). Enzymatic digestion was then performed using an SCS solution containing 10 mg/mL Lysing enzyme at 28 °C for 30 min. Following digestion, the cells were washed again with SCS and resuspended in STC (Tris-HCl 1% (v/v), CaCl2 1.1% (w/v), Sorbitol 18.2% (w/v)), yielding a protoplast suspension that was either stored at −80 °C or used directly for transformation. For PEG‑mediated transformation, the protoplasts were mixed with heparin sodium and two fusion fragments (LB and RB), incubated on ice for 10 min, after which 40% PEG3350 was added and the mixture was kept on ice for another 15 min. The mixture was then combined with YePS‑soft semi‑solid medium pre‑warmed to approximately 45 °C, immediately spread onto a selective regeneration plate containing hygromycin (bottom layer: YePSS solid medium with 200 μg/mL hygromycin; middle layer: YePSS solid medium without hygromycin), and cultured at 28 °C for 4–7 days until transformants appeared.

**
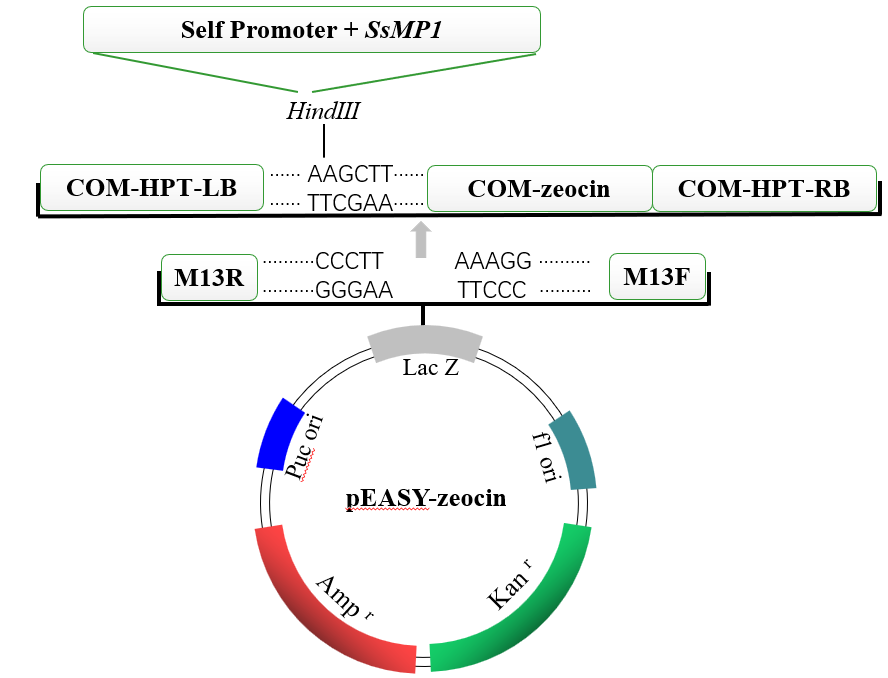
**

**Sequencing Results-**COM-HPT-LB-F/R

GCGCGCGTAATACGACTCACTATAGGGCGAATTGGGTACCGGGCCCCCCCTCGAGCGATCCTTGAAGCTGTCCCTGATGGTCGTCATCTACCTGCCTGGACAGCATGGCCTGCAACGCGGGCATCCCGATGCCGCCGGAAGCGAGAAGAATCATAATGGGGAAGGCCATCCAGCCTCGCGTCGAGCTTTGAAGTTGCTGCAAGCTGGCTTCAAGCCATCCCATCCGAATGTGATGGATGCGTTCTTTCTGGGCCGTTGCGACTTTGGGGATCGTCTTTCCCGCGCCCTTGGTTGGAGGCCCTGTCTCCGGTGTCCCTTGTCCCTTCCAGGCAAGCGAGCGAGGTCCATTCAGATGGTGCTCCATCAGCGTTGGCTTTCCGTCCCCATTGGCTCTTGGCAATTCGGTCAGCGGGGCTGACTGCCTCAGGTGGGGCAGTGCTAGTGTGTGTACCGACCCGCAGGATTGGTGCTTTGCCCAGAGCTCTACAGAATAGCGCGCGCATCCATATGTTAGTTCTGCAATTTTCTTGTATCGGTGCTGTGACTCATACTTCCCCCTTTGGCTGGCCTTGCGGCAACCAATAAGAACGCACAGTGAAATCTTGCGGGTGGGGAGTGGATCCATGGCGCCTGCATTGGCTTGGGGACGCGCACTGTCGCACACTTCCATCTGACCTTTCAGAAGGGTTTCGTGGTGGGCAAGGACCAACCGGTTGCGCGGCGTGCGTGGGTGCCTCGCCCGGCACTGCCAGGGCCACTGCAGTGGCAGTTTGCTGCCTGATACAAAATCCTTCCCTCCGCCCAGTTTTCCCTCTTTGACCTTCCTTTCTCTTCTCTGCAACCAAATCCACCCTATCAAACCAAAACAGTATCTCGACCGAGGTATCAACCTGAATCAGCAACATCGTAGCCAGCATTTGTCTCCGTCTCTGCAGAACCAGCGAGTTGCAAACATTATCCAGGCAACAGGGCACCAACTCACTTCTTCGGCTTTCACCAATCGGTACAGCTCTTCTCAGAACTCGCGTCCGCAACAGTTCTACGCTTCCTCAGCACCTTCTTCAGCTTCAATCCTGAACACTCAGAACCGCGCACAGCAGCGCCCTCCTGTTCCCTTGTTTCCCAAAAGTACCGGTAGTATTTCGCACGGAAAGCAGGGCAACAAGATGTTCTCAGGTACCCATATGAAAAAGCCTGAACTCACCGCGACGTCTGTCGAGAAGTTTCTGATCGAAAAGTTCGACAGCGTCTCCGACCTGATGCAGCTCTCGGAGGGTGAAGAATCTCGTGCTTTCAGCTTCGATGTAGGAGGGCGTGGATATGTCCTGCGGGTAAATAGCTGCGCCGATGGTTgatagtttaaactgaaggcgggaaacgacaatctgatccaagctcaagctaagctctcgatgccgcagtaggattgatggtggtgccacagcggagacgagagggtttccagccagcttgaagcggcgagcaagtttgactctggcatatccgactgatcgggcccgattccacaatgttcagctcacacatcggccgattattattattattattatttttttttttgggagccttccgcggctatttcttgctcggcgttttcttcgggccaagttttcataaaacgaagaaatctgcgaatggaaaaaatggggaaaaaaaatgccggtctgctgcacagtctggctcgcgtggaaaagcgcagcagctgtcaaaaaaaaaaaaaaaagcgtttacccgataagcaagcctgattctcgccgtctcagctcaacttgaacgccagcaacagggcaatcgagctggcttctcctctactgttatcggctaaagacagccagccgagcttcgagtcatgctcgggttctggtctcccggtgaagcgacgttcgacctcaagctctgcgtctaggtttggtatgcgtgagattcccaacaacaagcgatgcggaactcaaagacttggcattgatgccaccgcggaggatgagcccgttcttccgggttcgatgcacagctgcatctttgatcaaaggcgcttcgacgtttaacgttgtgcggttgtcctgtcttgttggtcgctgttggaaaagcaaaattgcccggcgaacacgccgaacgtcgtttgcagcagcatcttgttctttgcggagacgagttggtgatttcatgcaaaaacctgcatgcttccatcgcagtcgcagcatttcgcgtgggctccaaggggcgctagaggaggatcagagggcggccagacgttctaagcagttgctgcaaagttgacagcggcttcgggtgaattttccgcctgaaatcgtcttgtctatcaaaccaaatttgatcctctcttgagctttctcccaacctctcgggatgggtcaaccttgtcccatccatcgttagcatgggcttcccacgaccactgtggatttgtgtttgaacatactcacgcccatccttcctccttcacacgcacacattttgacgcaaacagcgttgcatgaaccgtaccgttcggacaagccgtgactcgttggtccccaccaaaaacataaaccttaccagcgcgctgtctctgcgcggctgcctctgcagtccggcttcatctcggcatctttccgacgcaaaagcaaccctcttttcttgtacagtcacaacgatcgagggtggcaaagcgcagcagcagtgtcgtttgactcagcctgctgcgaagaagactgccgtagtcagcgctttgatttgaacaataaaagagagactgactcagcttgtccccaccgcagcagcccctctttctttctttcagtcaaatggcgacatctttctaacctcatcacgccatacactttgaccttcagtcgcacatctccgttacacgctaacaattgaccccccccccccccccttcccttggccttatataatggacaagttcgagctcgacattctcgccaacgaggtcgaccagcaccgccgagcctcgctctcaccgagtcagatcgtcaatgatgatgcgaagaagcaacagatttgtgtcgacccggagaaggacgctctcgatacggtcgacagagcaaagtgcacgccagcgcttctgacctcgcagtcagcagacgatcaagacgtcgctaccaacaatgttgctccatccgagccggctgccgatgcagacacacaaccatccgatgacaccgacgagcccatcaaggaccgaggttgggcagcctggaagttcgtcctcgcctcggcggctaccgagttcatgatctggggcgcatcctacggctacggctccttccaagaataccaccagcacgatcccaactcgcccttccaccagacctcgctcaccgccacctcctccatcggcacctccctcctggcaggacagcatttcatcacgcttttcacgtttgggatctactcgatgtttccatcgctcatcaagatcttcacctacatctgtgtgatgggtgccgcgctttcgctgctcattgcgagcttcgccaactcggtggcgctgctgaccgtgttccagggtttgctgctgggtatgtttggaggaaacatcttcacgaccgtgatcttgtggctgcctgattggtgggatcagagacgtggctttgctaccgcgctcatctttgccggttctggggtgggtggaattctgtggccgatcatcttcacacagctgttgaccaaagtcggtttcagatggacccttcgcacatgggcgttgatccagctcatcgtgtccggcggtgcgacgctctgtctgcaaccaagccgcacaccgacacctctagcggcaccgctccgatggcgagccattctacctggtttcccacgctcgctcctctcaccagtgagtttgctcaacatcctcgcgctactccttcaaacaacagcttactattcggtggcgcttaatatctccaattacgcctcttcgatggggttcagcagcaccacctcgaccggcatcctctccgccttcaacgcctccgccgccatcacctacttcgtgctcggctacctcgtcgaccgcttcccctacccgctcctcatggccacctccaccgccctcaacctcgtcttcaccctgctcgtcttcggcttcgctggcgactccctcgccaagatcgtcatctacgtcgtgttttatgggttgaccgggggtgggtttagcagttttttaacgcctgtttcgagggatgcgtgggaggatggggggagggggtgtgagttttcgttgaggtttttgtatttggtgtgtgcgagggggttggcggcgatgttggggccgattgtggcggttcagttttatccggggaggttggggagggggagggggtgggggagttttgggtttagggggtttatagtgtttattgcggggacgttggcgttgtcgacgttggcgtcgttggcgatttttgcgtataagaagtgggggaagcagagggtgggggagggggtcaagaaggcggtgagtggtccggtgacgccgatggaggagggggggagggtggatggtggttgtgcggcgtgaggtgagactcgaatgtcacgcacacacgggggttcctgtgcaaaacgcatccacattccaacacattaaaactatacagaccgttctctctctactttttttcccgttggctggtgttcgtgaatcatatacggccattccacataccccttctccgccctctcgtcctcgaccctatctacaccaagcaccctaaacccagccttcaaataagcccccttccccatcgggctgctctccaaccacgcatccaccccttccttatccgccttcctcgtgccccacctgaccaacatcgtccccaaccccttcctctgaaatcccggatccaccaccaacagcttcaaaaaccaatacaccctcccctgcatcacaccttccctcgtccgattcatccttgcgaaaaacctgttcaacagcgtcggttcctcctccgctttcttatcgctttcctcgctgtctggtggtgtcctttcgtcgactagcttgattagatcttgctgataggcaggtttgcttggagaatggggggaaaagactgaccgaagaaacagcgagatctagaagtgataagcggaaagaatctgacttgctgtgatcagcagccaatttttttttcgtttttttttttcactccacatcgtcgtgcgtgcacggtctgcatgtgtaaattgtattcatcgaaagccacagttgaatacatcagcccgatgtggatttcgaaaaccaattaatcttggaattcacgcgctcagatcagtccatagagtcgacttcggctgtttccaagagcttcttctctgcgaggtggttgcccgtgtttctcgctgggaaaaaaggatcgattattattcgcttctacctcgctcgcacccttggcctgctgaaggaaacagcgccgagactcggtcacggttgctgggctccgtgttgatgctgggacggcgcaaagtggggcccgcgcactcttcgagccaaggacctcactcttcaagaacaagcgctgtcgccatcgtcttcttctttctgctccaccatcgaatctttctttctcgtttcgaaaccaaaacactcttccaccatggccaagttgaccagtgccgttccggtgctcaccgcgcgcgacgtcgccggagcggtcgagttctggaccgaccggctcgggttctcccgggacttcgtggaggacgacttcgccggtgtggtccgggacgacgtgaccctgttcatcagcgcggtccaggaccaggtggtgccggacaacaccctggcctgggtgtgggtgcgcggcctggacgagctgtacgccgagtggtcggaggtcgtgtccacgaacttccgggacgcctccgggccggccatgaccgagatcggcgagcagccgtgggggcaggagttcgccctgcgcgacccggccggcaactgcgtgcacttcgtggccgaggagcaggactgaactaGCCTGACCTATTGCATCTCCCGCCGTGCACAGGGTGTCACGTTGCAAGACCTGCCTGAAACCGAACTGCCCGCTGTTCTGCAGCCGGTCGCGGAGGCCATGGATGCGATCGCTGCGGCCGATCTTAGCCAGACGAGCGGGTTCGGCCCATTCGGACCGCAAGGAATCGGTCAATACACTACATGGCGTGATTTCATATGCGCGATTGCTGATCCCCATGTGTATCACTGGCAAACTGTGATGGACGACACCGTCAGTGCGTCCGTCGCGCAGGCTCTCGATGAGCTGATGCTTTGGGCCGAGGACTGCCCCGAAGTCCGGCACCTCGTGCACGCGGATTTCGGCTCCAACAATGTCCTGACGGACAATGGCCGCATAACAGCGGTCATTGACTGGAGCGAGGCGATGTTCGGGGATTCCCAATACGAGGTCGCCAACATCTTCTTCTGGAGGCCGTGGTTGGCTTGTATGGAGCAGCAGACGCGCTACTTCGAGCGGAGGCATCCGGAGCTTGCAGGATCGCCGCGGCTCCGGGCGTATATGCTCCGCATTGGTCTTGACCAACTCTATCAGAGCTTGGTTGACGGCAATTTCGATGATGCAGCTTGGGCGCAGGGTCGATGCGACGCAATCGTCCGATCCGGAGCCGGGACTGTCGGGCGTACACAAATCGCCCGCAGAAGCGCGGCCGTCTGGACCGATGGCTGTGTAGAAGTACTCGCCGATAGTGGAAACCGACGCCCCAGCACTCGTCCGAGGGCAAAGGAATAGAGTAGATGCCGACCGGGATCCACTTAACGTTACTGAAATCATCAAACAGCTTGACGAATCTGGATATAAGATCGTTGGTGTCGATGTCAGCTCCGGAGTTGAGACAAATGGTGTTCAGGATCTCGATAAGATACGTTCATTTGTCCAAGCAGCAAAGAGTGCCTTCTAGTGATTTAATAGCTCCATGTCAACAAGAATAAAACGCGTTTCGGGTTTACCTCTTCCAGATACAGCTCATCTGCAATGCATTAATGCATTGGACCTCGCAACCCTAGTACGCCCTTCAGGCTCCGGCGAAGCAGAAGAATAGCTTAGCAGAGTCTATTTTCATTTTCGGGAGACGAGATCAAGCAGATCAACGGTCGTCAAGAGACCTACGAGACTGAGGAATCCGCTCTTGGCTCCACGCGACTATATATTTGTCTCTAATTGTACTTTGACATGCTCCTCTTCTTTACTCTGATAGCTTGACTATGAAAATTCCGTCACCAGCCCCTGGGTTCGCAAAGATAATTGCACTGTTTCTTCCTTGAACTCTCAAGCCTACAGGACACACATTCATCGTAGGTATAAACCTCGAAAATCATTCCTACTAAGATGGGTATACAATAGTAACCATGGTTGCCTAGTGAATGCTCG

**Figure S3.** Schematic diagram of gene complementation.Using the genomic DNA of wild‑type strains *Ss16⁺* and *Ss16⁻* as templates, the primer pair *SsMP1*‑COM‑F/R was used to amplify fragments containing the promoter, terminator, and open reading frame of the *SsMP1* gene. The amplified fragments were then extracted from an agarose gel, and the recovered products were designated *SsMP1*‑COM and stored at –20 °C. The universal vector pEASY‑zeocin was digested with the restriction endonuclease HindIII (TransGen Biotech, JH101‑01) to obtain the linearized vector fragments, as described in the manufacturer’s manual. The vector fragments were also gel‑extracted and stored at –20 °C. Subsequently, the *SsMP1*‑COM fragment and the vector fragment were recombined and ligated using a rapid recombination cloning kit (Vazyme, C112‑01). Successful construction of the complementation vector pCOM‑*SsMP1* was confirmed using the primer pair *SsMP1*‑pCOM‑F/R. Using pCOM‑*SsMP1* as a template, two fragments, *SsMP1*‑HB‑F and *SsMP1*‑HB‑R, were amplified with the primer pairs COM‑HPT‑LB‑F/Zeocin‑R and Situ‑F/COM‑HPT‑RB‑R, respectively, and then gel‑purified. These two fragments were introduced into protoplasts of the*SsMP1* knockout mutant via polyethylene glycol (PEG)‑mediated protoplast transformation. Under the action of the endogenous homologous recombination machinery of *Sporisorium scitamineum*(sugarcane smut fungus), the *SsMP1* gene was restored. The only difference from the knockout procedure was that the bottom layer of the selective regeneration plates contained Zeocin at 100 μg/mL instead of hygromycin.


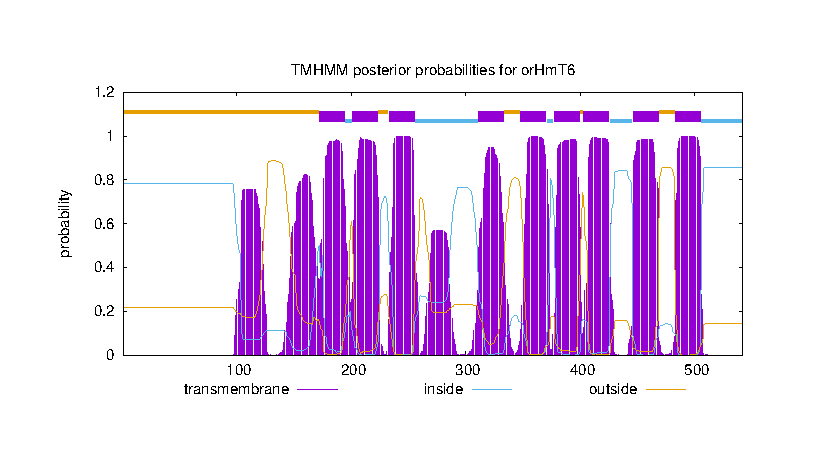


**Figure S4.** The predicted transmembrane helices of SsMP1


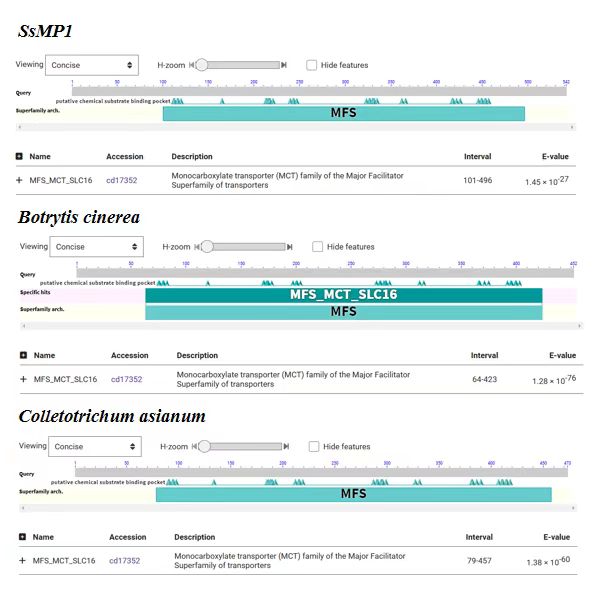


**Figure S5.** Conserved domain analysis between SsMP1 and MFS monocarboxylate transporters reported with role in pathogenesis in other fungi


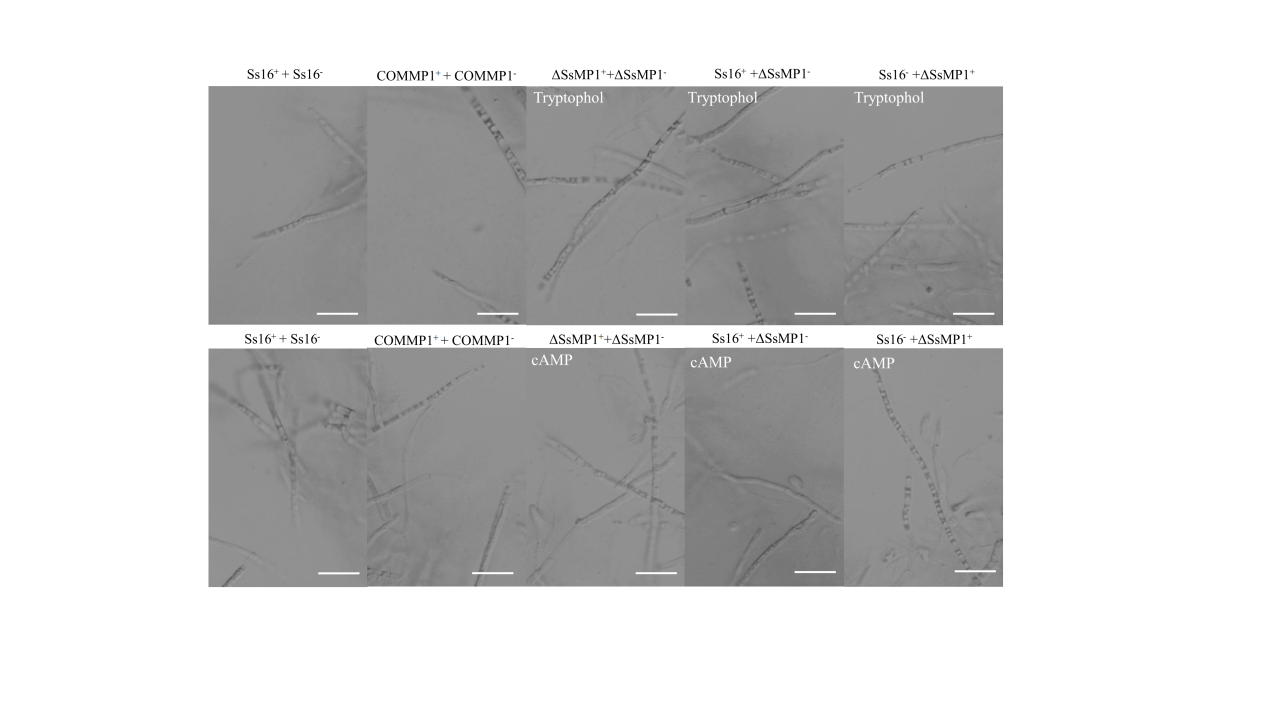


**Figure S6.** Microscopic images of the mycelium produced by the knockout mutant combination after restoring sexual mating ability with the addition of tryptophol or cAMP, as well as the mycelium produced by the wild-type combination under normal conditions and the complementary mutant combination (cultured at 28℃ in dark for 42 hours, observed under a 40×microscope), with a white bar representing 25 μm.


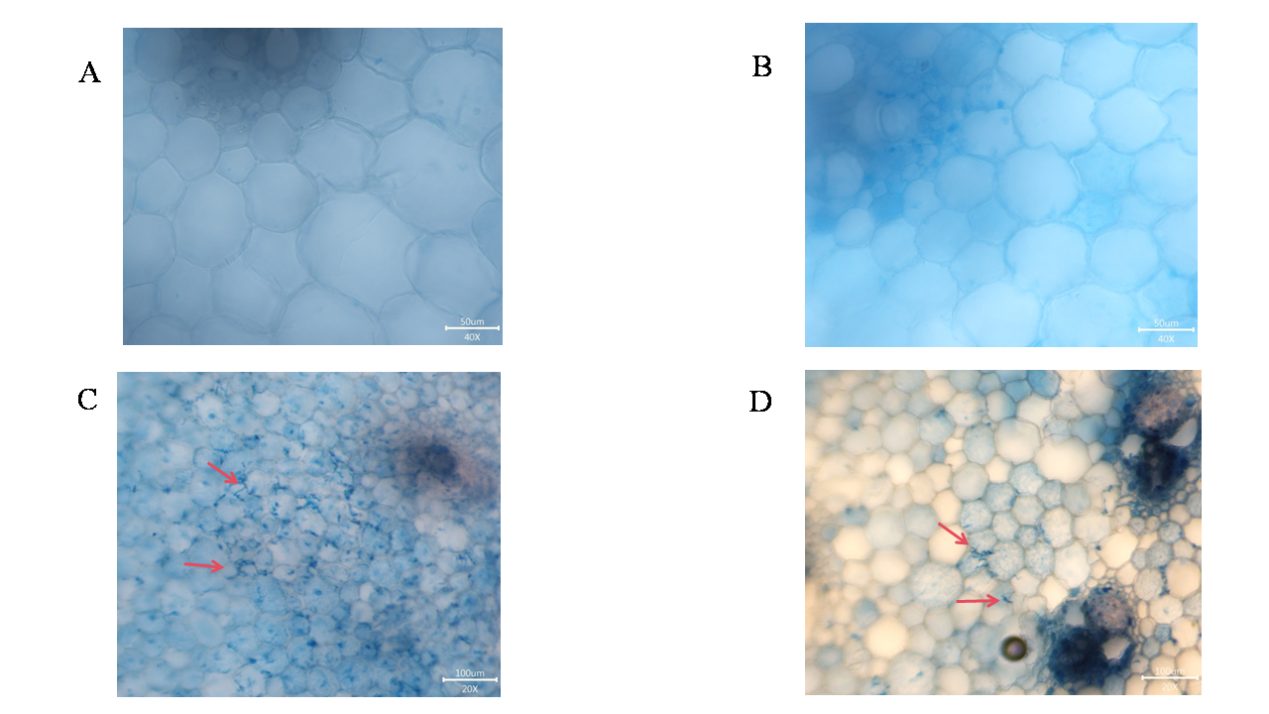


**Figure S7.** Visual observation of hyphae based on sugarcane stem apical meristem stained with 0.4% trypan blue.Red arrows indicate hyphae. No hyphaewas detected in sugarcane plants inoculated with sterile water or knockout mutant combination(*∆SsMP1^+^*+∆*SsMP1^−^*). Bar = 100 μm or 50μm. (A) sterile water, (B) knockout mutant combination(*∆SsMP1^+^*+∆*SsMP1^−^*), (C) wild-type combination(*Ss16^+^*+*Ss16^−^*), (D) complementary mutant combination (*COMMP1^+^*+*COMMP1^−^*).

**Total RNA extraction**

After homogenizing the sample with TRIzol reagent, chloroform was added to separate the homogenate into transparent upper water layer (containing RNA), middle phase and red lower organic layer (containing DNA and protein). RNA was precipitated from the water layer using isopropanol. Use ethanol to precipitate DNA from mesophase/organic layer. Protein was precipitated from phenol-ethanol supernatant by isopropanol precipitation. Wash the precipitated RNA, DNA or protein to remove impurities, and then resuspend to obtain RNA.

***SsMP1* gene sequence**

atggacaagttcgagctcgacattctcgccaacgaggtcgaccagcaccgccgagcctcgctctcaccgagtcagatcgtcaatgatgatgcgaagaagcaacagatttgtgtcgacccggagaaggacgctctcgatacggtcgacagagcaaagtgcacgccagcgcttctgacctcgcagtcagcagacgatcaagacgtcgctaccaacaatgttgctccatccgagccggctgccgatgcagacacacaaccatccgatgacaccgacgagcccatcaaggaccgaggttgggcagcctggaagttcgtcctcgcctcggcggctaccgagttcatgatctggggcgcatcctacggctacggctccttccaagaataccaccagcacgatcccaactcgcccttccaccagacctcgctcaccgccacctcctccatcggcacctccctcctggcaggacagcatttcatcacgcttttcacgtttgggatctactcgatgtttccatcgctcatcaagatcttcacctacatctgtgtgatgggtgccgcgctttcgctgctcattgcgagcttcgccaactcggtggcgctgctgaccgtgttccagggtttgctgctgggtatgtttggaggaaacatcttcacgaccgtgatcttgtggctgcctgattggtgggatcagagacgtggctttgctaccgcgctcatctttgccggttctggggtgggtggaattctgtggccgatcatcttcacacagctgttgaccaaagtcggtttcagatggacccttcgcacatgggcgttgatccagctcatcgtgtccggcggtgcgacgctctgtctgcaaccaagccgcacaccgacacctctagcggcaccgctccgatggcgagccattctacctggtttcccacgctcgctcctctcaccagtgagtttgctcaacatcctcgcgctactccttcaaacaacagcttactattcggtggcgcttaatatctccaattacgcctcttcgatggggttcagcagcaccacctcgaccggcatcctctccgccttcaacgcctccgccgccatcacctacttcgtgctcggctacctcgtcgaccgcttcccctacccgctcctcatggccacctccaccgccctcaacctcgtcttcaccctgctcgtcttcggcttcgctggcgactccctcgccaagatcgtcatctacgtcgtgttttatgggttgaccgggggtgggtttagcagttttttaacgcctgtttcgagggatgcgtgggaggatggggggagggggtgtgagttttcgttgaggtttttgtatttggtgtgtgcgagggggttggcggcgatgttggggccgattgtggcggttcagttttatccggggaggttggggagggggagggggtgggggagttttgggtttagggggtttatagtgtttattgcggggacgttggcgttgtcgacgttggcgtcgttggcgatttttgcgtataagaagtgggggaagcagagggtgggggagggggtcaagaaggcggtgagtggtccggtgacgccgatggaggagggggggagggtggatggtggttgtgcggcgtga

**Amino acid sequence (SsMP1)**

MDKFELDILANEVDQHRRASLSPSQIVNDDAKKQQICVDPEKDALDTVDRAKCTPALLTSQSADDQDVATNNVAPSEPAADADTQPSDDTDEPIKDRGWAAWKFVLASAATEFMIWGASYGYGSFQEYHQHDPNSPFHQTSLTATSSIGTSLLAGQHFITLFTFGIYSMFPSLIKIFTYICVMGAALSLLIASFANSVALLTVFQGLLLGMFGGNIFTTVILWLPDWWDQRRGFATALIFAGSGVGGILWPIIFTQLLTKVGFRWTLRTWALIQLIVSGGATLCLQPSRTPTPLAAPLRWRAILPGFPRSLLSPVSLLNILALLLQTTAYYSVALNISNYASSMGFSSTTSTGILSAFNASAAITYFVLGYLVDRFPYPLLMATSTALNLVFTLLVFGFAGDSLAKIVIYVVFYGLTGGGFSSFLTPVSRDAWEDGGRGCEFSLRFLYLVCARGLAAMLGPIVAVQFYPGRLGRGRGWGSFGFRGFIVFIAGTLALSTLASLAIFAYKKWGKQRVGEGVKKAVSGPVTPMEEGGRVDGGCAA

**Amino acid sequence (CgMCT1)**

MENVVRPPAVEYGAQAADPDQMTLTDNHYTHKQTNMLSNQEFQPYEPDQRHSEGQQSSSHTDEEKSEAIVDAPDGGFVGWLQVLSAFLLVLDGFGFITAFGVFQSFYVEELHGSTASDVSWIGSMQIFLLFLLGTVSGRAIDAGYFRTTLLTGFIFQIGGIFGASWSDRYWQLLLSQGIATGIGNGMHFTALVWLVSQYFTKKRGLALGISSCGAPIGAVIFTIMARQLIPAVGIAWTLRAMGFLVLFNSVIIFLISRPKETKRSSGPLLELAAFKELPYLLFTIGMFFTLLGAYFAYYYVPLFGRKNLGLDDNGALTIL

IIMSAVGITGRLIPPYFADRSIKPLRTLVISTLLSSLNVYAWIGVHSTTGLTVWVIAYAFTVNAVQTLFTASMGEVTSDMSKLGVRIGMVFTVVSFACLAGPPIGGSLVTLGKGNFLYAQLFAGTTMLVGGLLVALAKIKQVGQKDFWRITHS

**Amino acid sequence (MctP)**

MTERPKIRVLLIDNHPLVLDGLKAVLETFDHIEVAGTAGLAQTGLEIGRQVLPQVVLMDINMPKLSGIDAIELFRNELPQARVVMLSMHDSREYISSSVMHGAAGYILKDVSTDEIVSAIETVAGGGTYFSSGVFDALMGERAEEGSDPLTPRERDILGL

IVAGRSNKEIAETLGITSATTETHRKNLKKKLGIATTAGLIRYALDHGIVSKVG

**Amino acid sequence (BcMctA)**

MESTKTHKVKDGPTNIGIAVDSDVVIERSVEEAETKNERLEAVQSESEKQNAFIMDFPDGGARAWSVAAGAAGVLFCTFGYINAFGVYQEYYQTHQLSHRTPSDISWIGSLQVFFLFSGSAVGGPLFDRYGGRTIWPAALLYVFSVMMTSLCKEYYQFMLAQGILGGVATGMTMAPGMTAVGQYFNKKRGAAMGITVAGSSVGGVIFPIA

LAKMFANPKLGFGWSVRIIGFIMLAVLGISCTTIRARLPPRKKSFFLPAAFKELPYITLLFSAFLMILGVFIPIFYLPSYAVQYGMSTELASYLVSILNGASFFGRVVIPGILADKIGRLNMLCAVGVSTGILVFCWQSITSNAGIIVFAAIYGFCSGAIVSLVTLCLAMVPKNPQNIGTYMGMGMFVTAFAALIGPPINGALVERYHSFHQVADFSGVVVLVGGLSVLVIKVVEGGILKKV
